# Weighted gene co-expression network analysis reveals genes related to growth performance in Hu sheep

**DOI:** 10.1038/s41598-024-63850-x

**Published:** 2024-06-06

**Authors:** Qiang Wang, Jie Xu, Menghuan Bao, Huining Wang, XiaoMei Sun, Dejun Ji, Jian Wang, Yongjun Li

**Affiliations:** 1 Key Laboratory for Animal Genetics and Molecular Breeding of Jiangsu Province, College of Animal Science and Technology, Yangzhou University, Yangzhou, 225009 China; 2 International Joint Research Laboratory in Universities of Jiangsu Province of China for Domestic Animal Germplasm Resources and Genetic Improvement, Yangzhou University, Yangzhou, 225009 China

**Keywords:** RNA sequencing, WGCNA, Hu sheep, Growth performance, Slaughter performance, Meat quality, Functional clustering, Gene ontology, Gene regulatory networks

## Abstract

Hu sheep are a unique breed in our country with great reproductive potential, the extent of whose breeding has been steadily rising in recent years. The study subjects in this experiment were 8-month-old Hu sheep (n = 112). First of all, the growth performance, slaughter performance and meat quality of their eye muscle quality were assessed, meanwhile their live weight, carcass weight, body length, body height, chest circumference, chest depth and tube circumference were respectively 33.81 ± 5.47 kg, 17.43 ± 3.21 kg, 60.36 ± 4.41 cm, 63.25 ± 3.88 cm, 72.03 ± 5.02 cm, 30.70 ± 2.32 cm and 7.36 ± 0.56 cm, with a significant difference between rams and ewes (*P* < 0.01). Following that, transcriptome sequencing was done, and candidate genes related to growth performance were identified using the weighted co-expression network analysis (WGCNA) approach, which was used to identified 15 modules, with the turquoise and blue modules having the strongest association with growth and slaughter performance, respectively. We discovered hub genes such as ARHGAP31, EPS8, AKT3, EPN1, PACS2, KIF1C, C12H1orf115, FSTL1, PTGFRN and IFIH1 in the gene modules connected with growth and slaughter performance. Our research identifies the hub genes associated with the growth and slaughter performance of Hu sheep, which play an important role in their muscle growth, organ and cartilage development, blood vessel development and energy metabolic pathways. Our findings might lead to the development of potentially-useful biomarkers for the selection of growth and slaughterer performance-related attributes of sheep and other livestock.

## Introduction

China has a large-scale animal husbandry industry and a massive source of sheep genetics, accounting for around 10% of the world's 700 sheep types. In our country, the meat production performance of sheep breeds is currently far from the optimum. Lamb growth rates and feed conversion rates are two factors that affect sheep economic output.

Hu sheep are a unique native Chinese sheep breed and one of the world's few white types, which possesses excellent adaptability, precocity, fertility, breastfeeding performance, quick growth and development, as well as resistance to extreme temperatures and humidity. Hu sheep are descended from grazing Mongolian sheep, which have been bred for over 800 years in Jiangsu and Zhejiang. The majority of Hu sheep are raised in Jiaxing, Zhejiang, and the Taihu Lake region. In Xinjiang, Gansu, Linxia, and other places of China, they are being introduced and raised, which have been referred to as the best sheep breed for large-scale breeding.

There is yet to be a systematic examination on the regulatory mechanisms of the skeletal muscle growth and development in postnatal animals. In recent years, the fast development of next-generation sequencing technologies and bioinformatics approaches such as RNA-seq has enabled circumstances for exploring regulatory networks that regulate Hu sheep growth and development, such as skeletal muscle development and fat deposition.

Understanding the complex network structure and evaluating whether or not there are hub genes may be performed by examining gene expression variations and complex interaction patterns among genes. Previously, a systems biology approach named weighted gene co-expression network analysis (WGCNA) was used to study hub genes playing important roles in gene modules^1^. WGCNA can be used to define correlation patterns across a wide range of transcript measurements across several microarrays, whereas traditional methods are used to compare matched values. WGCNA was developed to find modules/clusters of highly-correlated genes and connect modules to quantitative factors, which has recently been utilized effectively in a number of studies^2–4^.

The growth performance, slaughter performance and meat quality traits of the eye muscle of 8-month-old Hu sheep were measured in this study. Growth performance includes live weight, body length, body height, chest circumference, chest width, chest depth and tube girth. Slaughter performance includes carcass weight, eye muscle area, and backfat thickness. WGCNA was used to identify modules closely related to the growth, development, and slaughter performance of Hu sheep in this study, enrichment pathways within key modules were analyzed, and hub genes were screened out, which might be potential candidate genes for Hu sheep breeding.

## Materials and methods

### Ethics statement

This study was approved by the Institutional Animal Care and Use Committee (IACUC) of the Yangzhou University Animal Experiments Ethics Committee (Permit Number: SYXK (Su) IACUC 2012-0029). All animal experiments were conducted in accordance with national guidelines. The design and implementation of experiments were carried out in accordance with ARRIVE guidelines.

### Animal feeding and management

Jiangsu Qianbao Animal Husbandry Co., Ltd. supplied 8-month-old Hu sheep, all of which had the same genetic background, feeding circumstances and management settings and were weaned at 2 months of age.

### Growth performance and slaughter performance and meat quality determination

**Figure 1:**
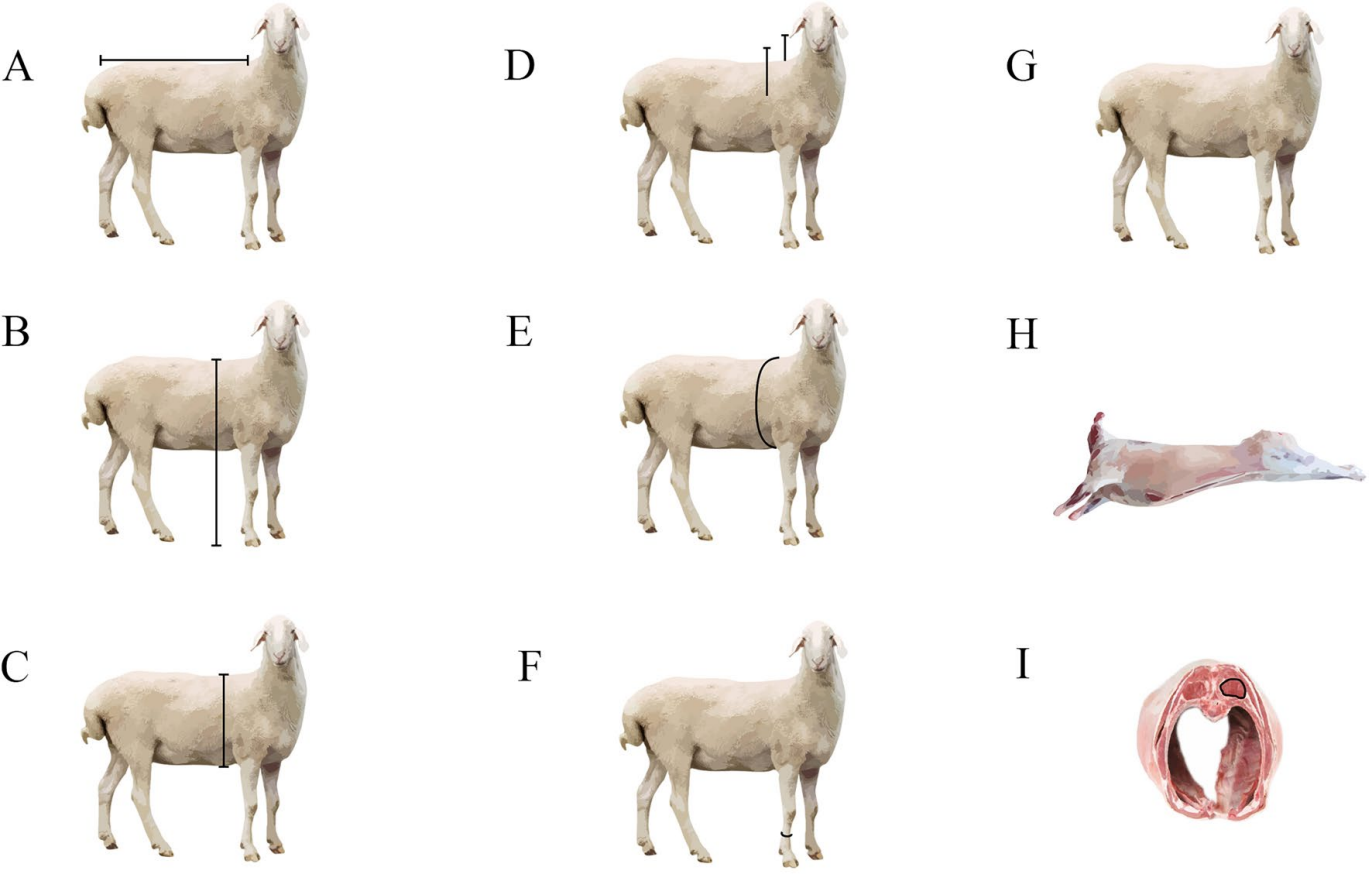
Schematic diagram of growth performance and slaughter performance (**A**: body length, **B**: body height, **C**: chest depth, **D**: chest width, **E**: chest circumference, **F**: tube circumference, **G**: live weight, **H**: carcass weight, **I**: back fat thickness and eye muscle area).

After fasting for 24 h before slaughter, the growth performance of the Hu sheep was measured on the day of slaughter, including live weight (Fig. 1G), body length (Fig. 1A), body height (Fig. 1B), chest circumference (Fig. 1E), chest width (Fig. 1D), chest depth (Fig. 1C) and tube circumference (Fig. 1F), using the technique of assessing the production performance of reference sheep. When measuring, the Hu sheep should stand on a flat place with an upright posture. The measurer usually stands on one side of the Hu sheep and remains quiet while measuring. Measuring tools should be close to the surface of the measuring part.

All sheep are killed through traditional neck cutting and dehaired as soon as possible after slaughter. Within 30 min of slaughter, back fat thickness (Fig. 1I) and eye muscle area (Fig. 1I) measured with B-ultrasound (Echo Blaster 128, Beijing Bevic Co, Ltd, Beijing, China) at the position of 3 cm behind the last rib from the midline, and an acidometer (PH-STAR, Matthaus, German) was used to determine the pH value of eye muscles.

After weighing the carcass (Fig. 1H), the eye muscles are separated from the part behind the last rib and bisected. One piece was chilled at 4 °C and returned to the laboratory within 4 h, with its shear force, water loss rate, and meat composition assessed. Another fragment was frozen in liquid nitrogen in preparation for a gene expression investigation.

The shear force was quantified using a digital tenderness meter (Bosin-BS, BosinTech, China)^5^. Then, the water loss rate was determined using the pressure method^5^. The meat composition of the eye muscles, including their moisture content, protein content, fat content, and collagen content, was measured using a FoodScan analyzer (FoodScan Laboratory, UK) through near-infrared transmission spectroscopy.

### Extraction of RNA and analysis of cDNA libraries

For the purpose of isolating total RNA from muscle tissues, we used an animal tissue total RNA extraction kit (Tiangen, Beijing, China) and following the instructions supplied by the vendor. We used a NanoDrop 8000 spectrophotometer (NanoDrop, Waltham, MA) to measure the RNA concentration and employed agarose gel electrophoresis to measure its purity. The RNA integrity was assessed using a 2100 bioanalyzer from Agilent Technologies, located in Santa Clara, CA. Shenzhen Huada Genome Co., Ltd. (located in Shenzhen, China) conducted high-throughput RNA sequencing and deep sequencing procedures.

### Aligning sequenced reads to reference genomes

The filter module of ***SOAPnuke***^6^ (https://github.com/BGI-flexlab/SOAPnuke) (version "1.5.3") software was used to preprocess FASTQ files, including trimming (adapter and low quality end, etc.) if set, discarding (adapter, low quality, high N base ratio and etc.) and generating a statistic report. The filtered “clean reads” were saved in the ***FASTQ*** format. The ***Hisat2***^7^ (https://daehwankimlab.github.io/hisat2/) (version "2.2.1") program was used for sequence alignment between the preprocessing sequence and the latest sheep reference genome (***ARS-UI_Ramb_v2.0***) of each sample. Then ***Samtools***^8^ (http://www.htslib.org/) (version "1.7–2") was used to convert the sam files generated by Hisat2 into bam files, and which were sorted using the sort function. The gene expression level of each sample was then calculated using the ***Stringtie2***^9^ (https://github.com/skovaka/stringtie2) (version "2.2.1") software. Sequence similarity comparison and ***FPKM*** value were used to quantify the gene expression abundance of each sample.

### Utilizing a method called weighted gene co-expression network analysis

The development of a co-expression network was carried out in this investigation using the R package ***WGCNA***^10^ (https://cran.r-project.org/web/packages/WGCNA/index.html) (version "1.72–1"). The second study was focused on the top 5000 expressed genes, chosen based on their median absolute deviation (***MAD***), so as to assure the heterogeneity and correctness of their bioinformatics for a co-expression network analysis of 112 samples. The ***blockwiseModules*** function of the WGCNA package was employed to construct a co-expression network for a single step. The parameters were set as follows: ***minModuleSize*** = 100, power = 12 (with a correlation coefficient threshold of 0.85), ***TOMType*** = "signed", ***mergeCutHeight*** = 0.35 (to merge potentially-similar modules), ***verbose*** = 3, and ***maxBlockSize*** = 30,000. Next, we conducted a correlation analysis between the phenotypic and gene expression data using the ***labeledHeatmap*** function. This analysis allowed us to identify strongly-associated co-expression modules, using a screening requirement of ***P*** < 0.01. Since WGCNA generated a huge gene network, genes in each module were imported into ***Cytoscape***^11^ (https://cytoscape.org/) (version "3.10.0") using the ***exportNetworkToCytoscape*** function (threshold = 0.02, weighted = TRUE) to construct a co-expression network. The top 300 were selected according to the weight value to further narrow the network, locate the central gene, and visualize the gene relationship.

### Genes inside functional enrichment analyses of co-expression modules

Following the identification of modules with a high degree of association with the phenotypes, a GO analysis and a KEGG pathway analysis^12^ (https://www.kegg.jp/kegg/kegg1.html) were conducted. The ***ClusterProfiler***^13^ (https://bioconductor.org/packages/release/bioc/html/clusterProfiler.html) (version "4.12.0") R program was used to detect significant biological processes linked to genes inside a module and conduct gene cluster enrichment analyses. The ***AnnotationHub*** (https://www.bioconductor.org/packages/release/bioc/html/AnnotationHub.html) (version "3.12.0") (hub: AH107722) was used to annotate the gene ontology (GO) entry for sheep. The ***Ensembldb***^14^ (https://bioconductor.org/packages/release/bioc/html/ensembldb.html) (version "2.28.0") package was used to annotate gene information. The GO word analysis included the following parameters as the cutoff criteria: ***p-value***: 0.05; ***q-value***: 0.2. The KEGG pathway enrichment study included the following parameters as cutoff criteria: ***organism***: OAS; ***p-value***: 0.05; ***q-value***: 0.2. The data was shown using the R package ***GOplot***^15^ (https://wencke.github.io/) (version "1.0.2").

## Statistical analysis

The phenotypic values of each growth trait of Hu sheep were presented as the mean ± standard deviations (mean ± SD). The R function t.test to conduct ***t-tests*** with a paired parameter set to False to analyze the differences between rams and ewes. Phenotypic data was visualized using the *ggplot2*^16^ (https://ggplot2.tidyverse.org/) (version "3.5.1") package, using geom_smooth to create a simple linear model and plot that modeled over the data. To do this, we would set method = 'lm'. Correlation coefficients among phenotypes were calculated and visualized using the *Hmisc*^17^ (https://cran.r-project.org/web/packages/Hmisc/index.html) (version "5.1–2") package.

## Results

### Summary of growth performance a slaughter performance and meat quality

**Figure 2:**
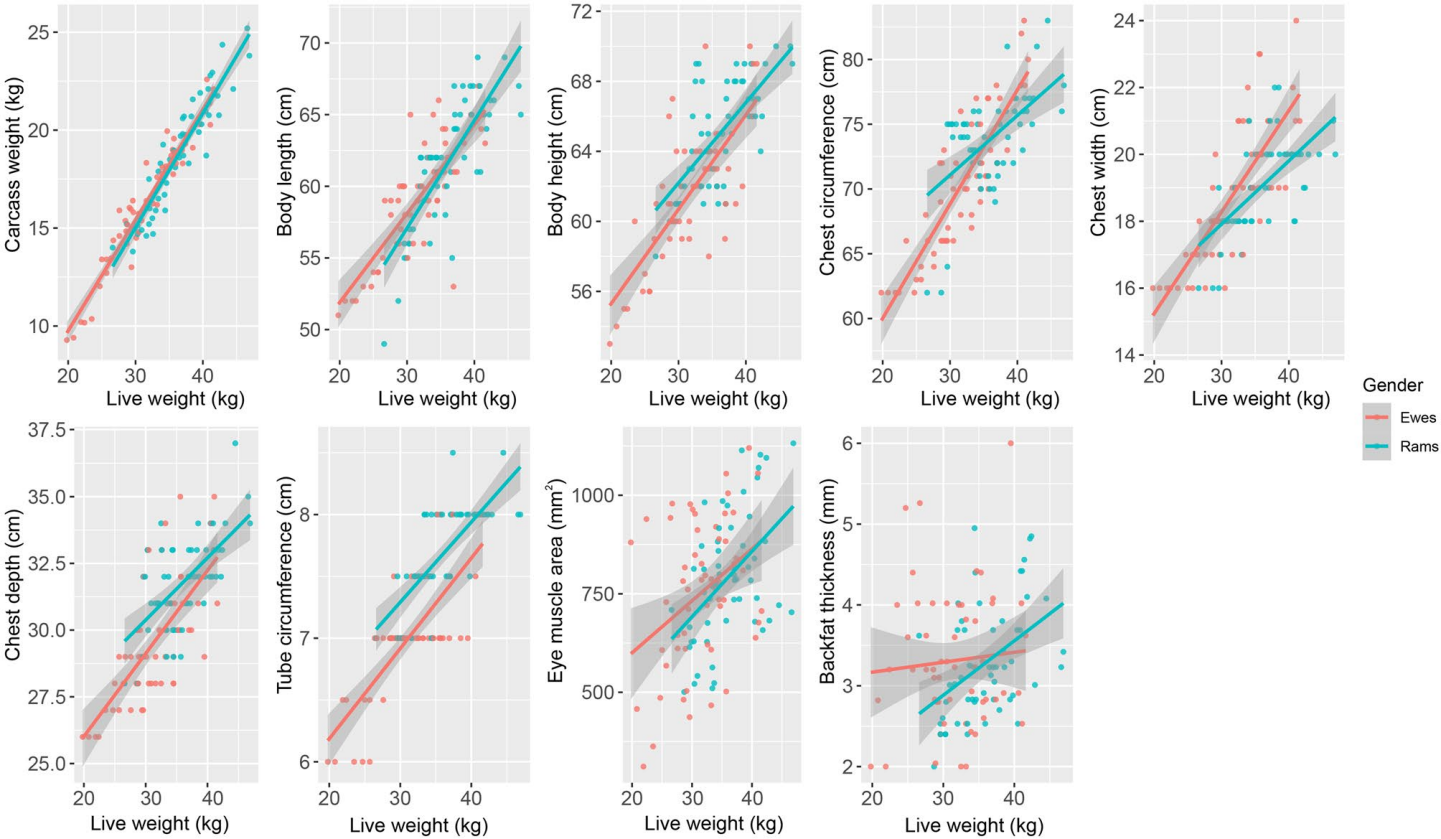
Summary statistics of live weight with other 9 growth performance and slaughter performance (carcass weight, body length, body height, chest circumference, chest width, chest depth, tube circumference, eye muscle area, backfat thickness).

Figure 2 and Table 1 show the summary statistics of live weight with other 9 growth and slaughter performance; these indicators increased with the increasing live weight while exhibited distinct variations between rams and ewes. The average live weight of the 8-month-old Hu sheep (ram = 56, ewe = 56) was 33.81 ± 5.47 kg, and the carcass weight was 17.43 ± 3.21 kg. The average live weight, carcass weight, body length, body height, chest circumference, chest width, and chest depth of the rams were significantly higher than those of the ewes (***P*** < 0.01); chest width and eye muscle area were larger than those of ewes, but did not show a significant difference (***P*** > 0.05), backfat thickness of ewes was greater than that of rams, but not at a significant level (***P*** > 0.05).

**Table 1:** Summary of growth performance and slaughter performance and meat quality performance of the 8-month-old Hu sheep.

	Trait	Average value	Average value (Rams)	Average value (Ewes)
Body size performance	Live weight, kg	33.81 ± 5.47	36.12 ± 4.62 a	31.50 ± 5.23 b
	Body length, cm	60.36 ± 4.41	61.68 ± 4.32 a	59.04 ± 4.05 b
	Body height, cm	63.25 ± 3.88	65.00 ± 3.05 a	61.50 ± 3.79 b
	Chest circumference, cm	72.03 ± 5.02	73.89 ± 3.80 a	70.16 ± 5.34 b
	Chest width, cm	18.90 ± 1.74	19.07 ± 1.36	18.73 ± 2.03
	Chest depth, cm	30.70 ± 2.32	31.80 ± 1.75 a	29.59 ± 2.27 b
	Tube circumference, cm	7.36 ± 0.56	7.69 ± 0.41 a	7.03 ± 0.49 b
Slaughter performance	Carcass weight, kg	17.43 ± 3.21	18.60 ± 2.88 a	16.25 ± 3.07 b
	Eye muscle area, mm2	772.62 ± 175.92	793.30 ± 161.05	751.94 ± 185.88
	Backfat thickness, mm	3.30 ± 0.78	3.29 ± 0.70	3.31 ± 0.84
Meat quality performance	pH	5.69 ± 0.49	5.65 ± 0.51	5.72 ± 0.45
	Shear force, N	50.70 ± 12.00	51.99 ± 12.50	49.40 ± 11.23
	Water loss rate, %	0.24 ± 0.05	0.24 ± 0.06	0.24 ± 0.05
	Moisture content, %	75.48 ± 1.29	75.49 ± 1.07	75.47 ± 1.46
	Protein content, %	20.65 ± 0.95	20.61 ± 0.94	20.68 ± 0.96
	Fat content, %	2.86 ± 1.15	2.83 ± 1.07	2.90 ± 1.21
	Collagen content, %	0.58 ± 0.29	0.58 ± 0.29	0.59 ± 0.28

a–b Means within a row with different subscripts differ when ***P*** value < 0.01.

Among meat quality, pH (5.69 ± 0.48), shear force (50.82 ± 12.01, N), water loss rate (0.24 ± 0.05, %), moisture content (75.49 ± 1.27), protein content (20.62 ± 0.94, %), fat content (2.91 ± 1.16, %) and collagen content (2.91 ± 1.16, %), the indicator worth noting was the fat content, which was larger in ewes than in rams (***P*** > 0.05), and there were no significant differences between the rams and ewes of other indicators.

We examined the correlation coefficients among growth performance, slaughter performance and meat quality performance of 8-month-old Hu sheep (Fig. S1). There is a significant correlation within these growth and slaughter performance, especially between live weight and carcass weight; live weight has the highest correlation with carcass weight (r = 0.96***), followed by that between live weight and body length (r = 0.82***). We also found the strongest negative correlation between fat content and moisture content (r = − 0.55***), followed by that between backfat thickness and moisture content (r = − 0.33***).

### An outline of RNA sequencing data

The clean data on each sample was around 7.0 GB, and the percentage of Q20 bases was greater than 95.32%. The percentage of clean reads mapped to the sheep reference genome ranged from 94.04 to 98.76%. 92.89% of pure readings were uniquely mapped and utilized for further investigations (Table S1).

### WGCNA analysis

**Figure 3:**
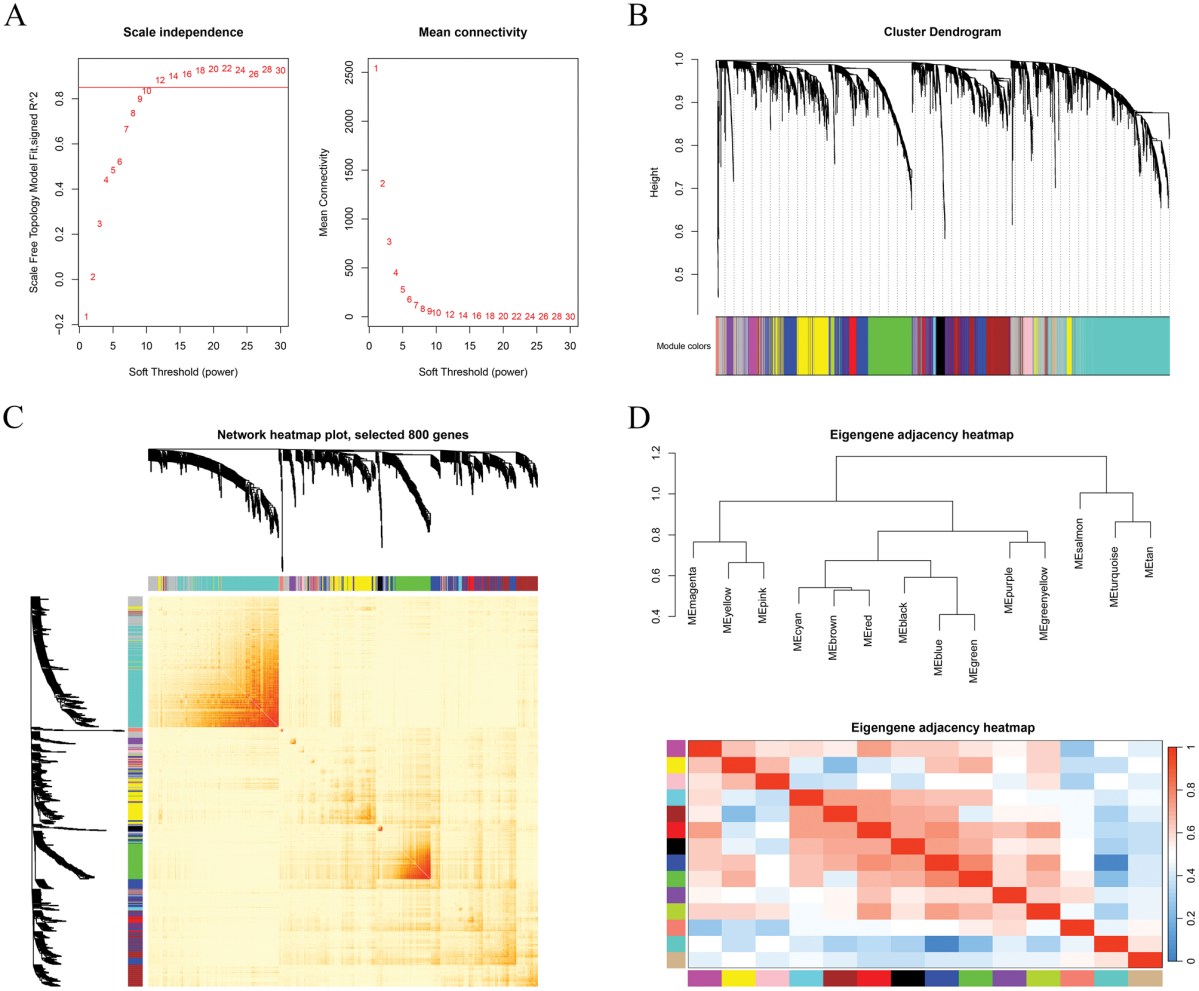
The procedure of screening genes for several traits of Hu sheep utilizing WGCNA, included growth performance, slaughter performance, and meat quality. Various soft-threshold powers are analyzed for network topology (**A**). A soft threshold of β = 12 was used to define the adjacency matrix, and scale-free topologies were investigated. (**B**) Gene cluster dendrograms using certain module colors and variations in topological overlap. In (**C**), we can see a heat map representing the co-expression module-based intergenic topological overlap matrix (TOM). A more vibrant backdrop suggests a stronger link among modules. (**D**) Heatmaps for gene network visualization.

After identifying and removing outlier samples using clustering, we proceeded to create a network. The co-expression network was based on the final expression matrix, which included 5,000 genes and 111 samples (Fig. S2). Setting the correlation coefficient as 0.85 causes a significant decrease in the average connectivity and a near match to the highest value of the scale-free topology model. Figure 3A shows that 8.5 is the right soft threshold. Out of the 15 modules produced, the turquoise module had the most genes, whose number was 1,114 (Fig. 3B) when the ***mergecutheight*** parameter and the ***minModuleSize*** parameter were set as 0.35 and 100. Supplementary Table S2 displays the gene counts of each module. Then, the interplay among these co-expression modules was examined using the Pearson correlation coefficient. In a cluster analysis, the modules underwent hierarchical eigengene clustering. Then, the branches (meta-modules) of the dendrogram were categorized according to the correlation among the eigengenes (Fig. 3C). Figure 3D shows a topologically overlapping heat map with various colors representing distinct gene clusters inside each module. Red indicates a positive association and blue indicates a negative correlation.

### Module–trait relationship analysis

**Figure 4:**
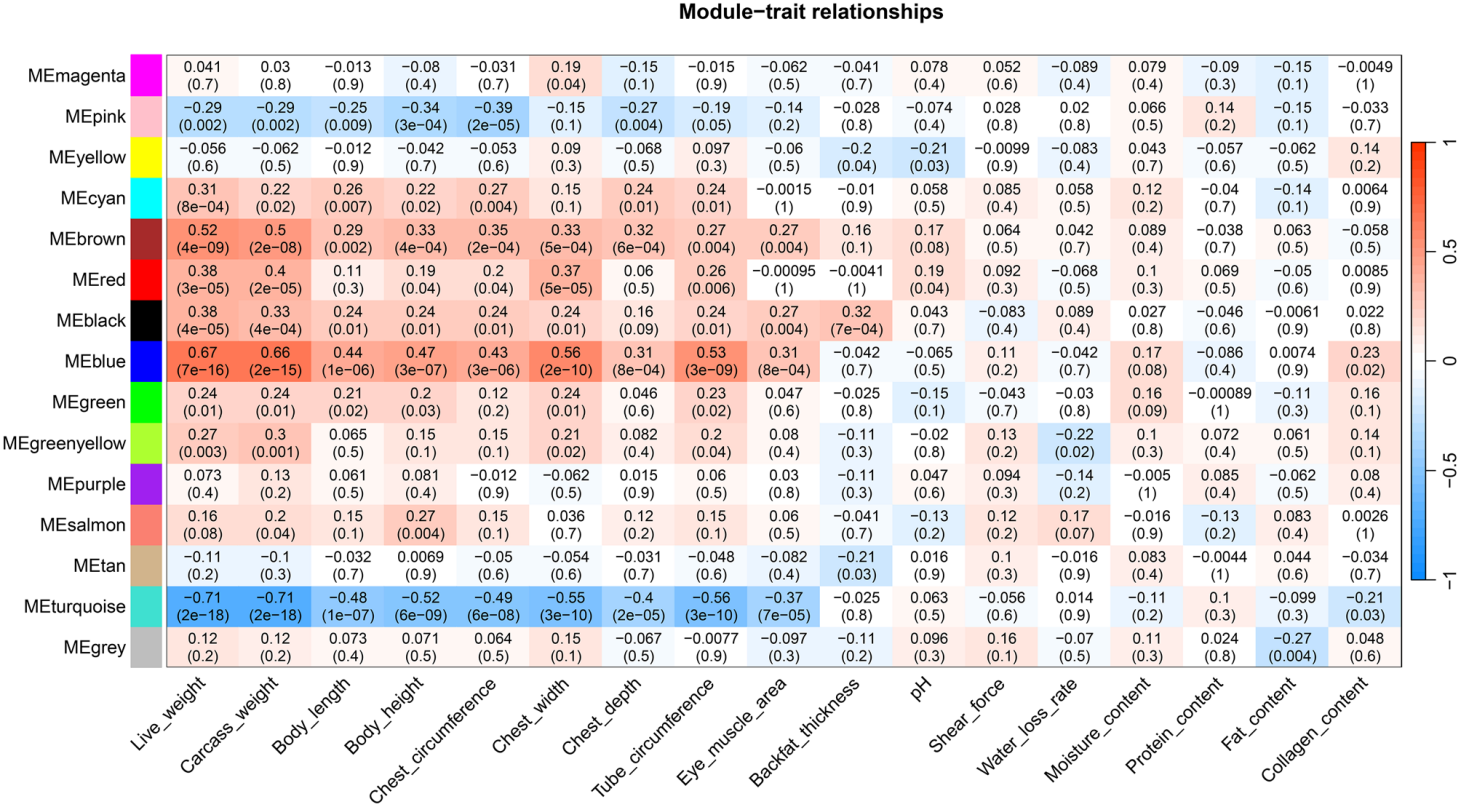
Analysis of the link between modules and traits. Each row represents a specific gene (eigengene) associated with a module, and each column represents a certain attribute. Every cell includes a related correlation and p-value.

Then, we performed a correlation analysis on 17 traits with the co-expression modules, and finding that the cyan, brown, red, black and blue modules have extensive positive correlations with slaughter performance (live weight, carcass weight, body length, body height, chest circumference, chest width, chest depth, tube circumference, eye muscle area and backfat thickness). Live weight and carcass weight were strongly positively correlated with blue and brown modules, with a correlation coefficient of ***r*** ≥ 0.5 and a significance of ***P*** ≤ 0.001 (Fig. 4). At the same time, it was found that live weight and carcass weight had a strong negative correlation with the turquoise module, whose correlation coefficient was*** r*** = − 0.71, and the significance was ***P*** ≤ 0.001 (Fig. 4).

A correlation study was conducted on gene significance (GS) and module membership (MM) to examine their connection, and the findings demonstrated a strong link between them (Fig. S3). Meanwhile, the GS of the turquoise and blue module might cause large variations in slaughter performance (live weight, carcass weight, body length, body height, chest circumference, chest width, chest depth, tube circumference, eye muscle area and backfat thickness).

### Functional enrichment analysis of critical modules

**Figure 5:**
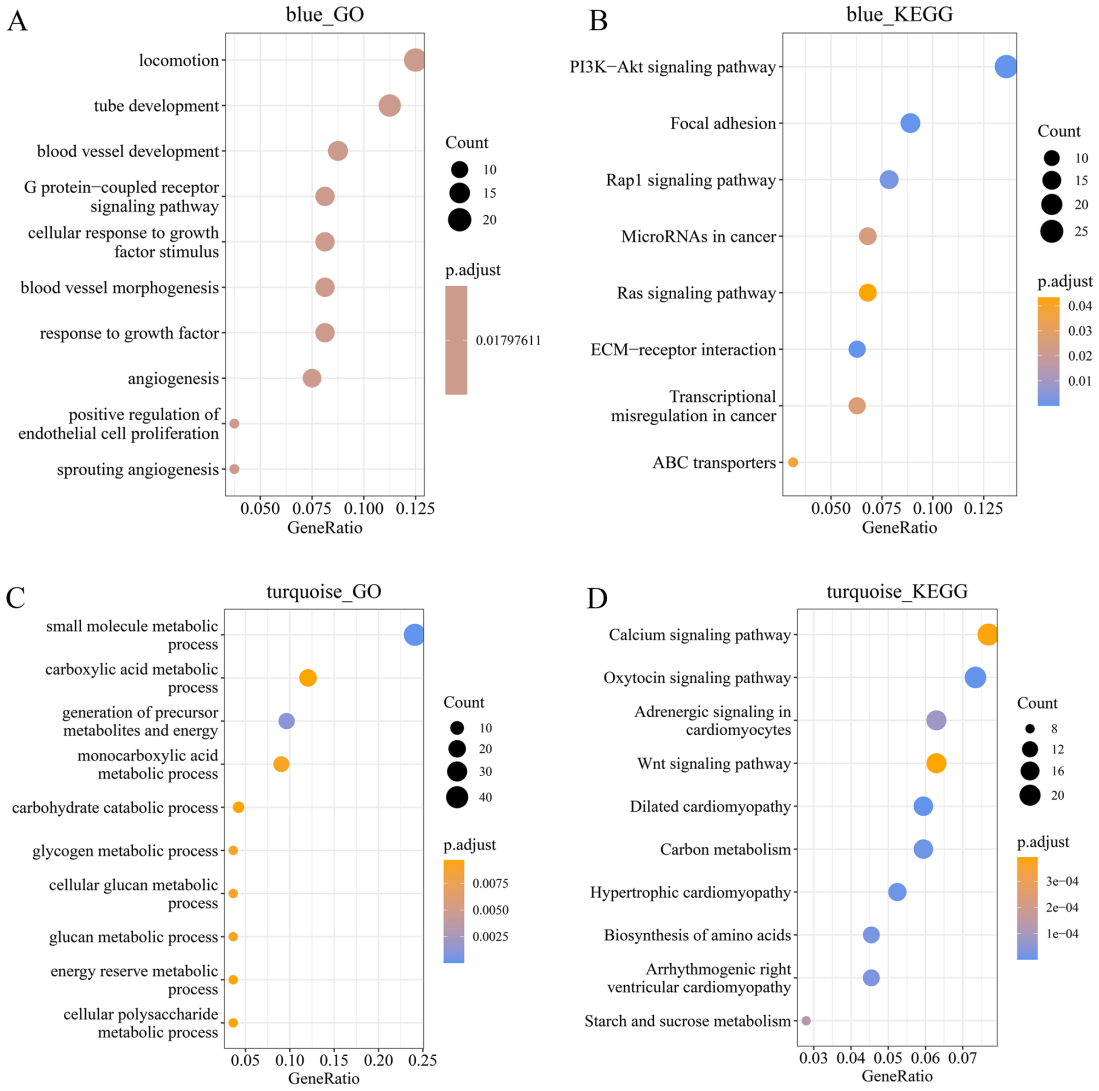
Functional annotation analysis of genes within the turquoise and blue modules. (**A**) The top 10 significantly-enriched GO terms of the genes in the blue module are shown. (**B**) The top 10 significantly-enriched KEGG pathways of the genes in the blue module are shown. (**C**) The top 10 significantly-enriched GO terms of the genes in the turquoise module are shown. (**D**) The top 10 significantly-enriched KEGG pathways of the genes in the turquoise module are shown.

We used functional enrichment analysis (Table S3) to learn more about the module genes and the biological processes they were involved in Fig. 5 and Table S3 show that there are more than 40 genes enriched in the turquoise module, and the findings demonstrate that the "small-molecule metabolic process" is considerably enriched in terms of BP. In addition, the genes in this module are also enriched in energy metabolism items such as carbohydrate catabolism, glycogen metabolism, and glucan metabolism. The genes in the blue (Fig. 5, Table S3) and brown (Fig. S4, Table S3) modules are enriched to tube development, blood vessel development, cellular responses to growth such as vascular system development and growth factor responses.

Genes in the red module (Fig. S4, Table S3) were enriched to the items of tissue development, organ development, skeletal system development, cartilage development, and connective tissue development. Genes in black (Fig. S4, Table S3) modules were enriched for defense response entries, including defense responses to other organisms and viruses, which were enriched in the positive production of interferon regulatory entries.

KEGG enrichment results showed that genes in the blue module (Fig. 5B, Table S3) were enriched in the PI3K-Akt signaling pathway, ECM-receptor interaction, focal adhesion and the Rap1 signaling pathway, etc. Genes in the turquoise (Fig. 5D, Table S3) module significantly enriched in the calcium signaling pathway, the oxytocin signaling pathway, as well as pathways related to heart function and energy metabolism related to starch and sucrose. Genes in the turquoise (Fig. 5D, Table S3) module were significantly enriched in the calcium signaling pathway, the oxytocin signaling pathway as well as pathways related to heart function and energy metabolism related to starch and sucrose. Genes in the pink module (Fig. S4, Table S3) were enriched in the cAMP signaling pathway and adrenergic signaling in cardiomyocytes. Genes in the cyan module (Fig. S4, Table S3) were enriched in motor proteins, cell cycle, human T-cell leukemia virus 1 infection. Genes in the brown module (Fig. S4, Table S3) were enriched in the MAPK signaling pathway. Genes in the red module (Fig. S4, Table S3) were enriched in protein digestion and absorption as well as ECM-receptor interaction. Genes in the black module (Fig. S4, Table S3) were enriched in human papillomavirus infection and measles.

### Hub gene selection

**Figure 6:**
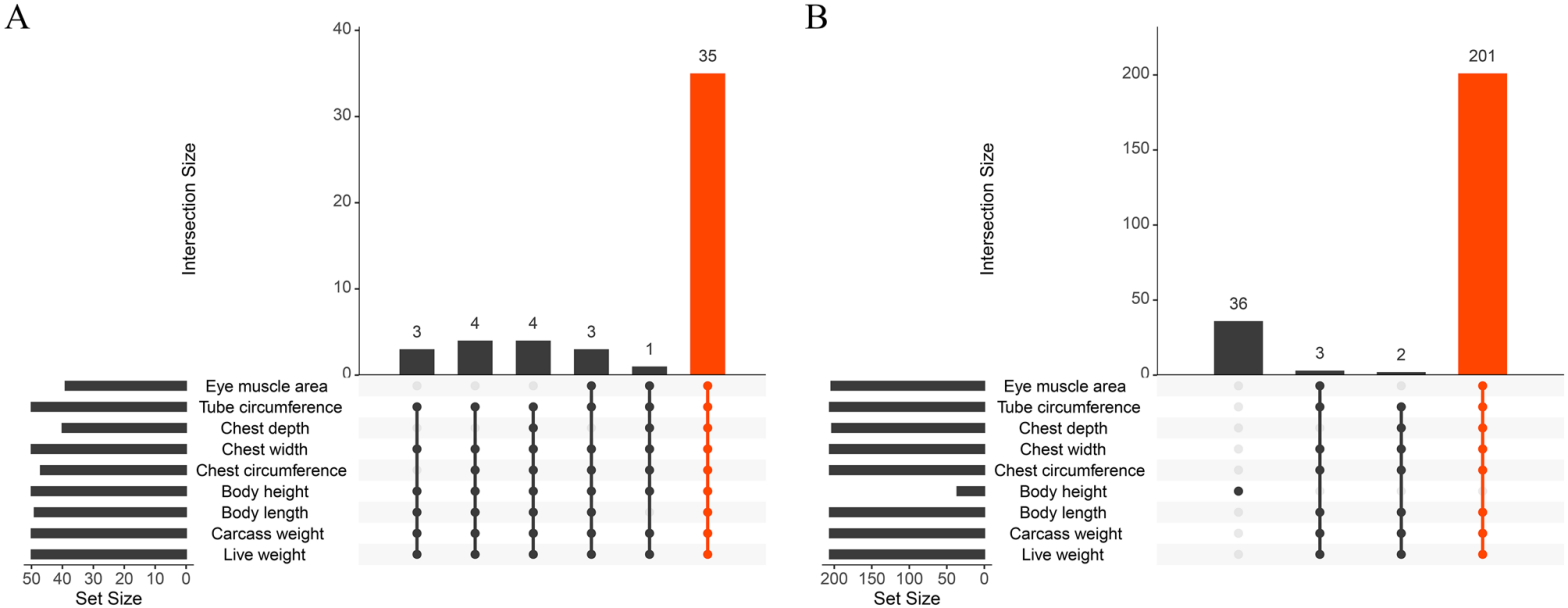
Genes that have common characteristics related to slaughter performance. The y-axis depicts the degree of overlap, while the dot on the x-axis represents the genes inside the associated gene set. The "set size" in the lower left corner denotes the number of genes in the linked gene set. The red dot signifies the presence of overlaps in slaughter performance.

Through the use of a WGCNA analysis, we have identified genes substantially associated with each module, which were picked based on their gene significance (GS) value being more than or equal to 0.2 and their module membership (MM) value being greater than or equal to 0.8. The purpose of this selection was to pinpoint genes specifically connected to growth and development. Figure 6A and B show the intersection results of all genes in the slaughterer performance traits (live weight, carcass weight, body length, body height, chest circumference, chest width, chest depth, tube circumference, eye muscle area) in the blue and turquoises, Fig. S5 shows the intersection results of all genes in pink, cyan, brown, red and black respectively. Then Ensembldb was applied to annotate gene information, and the results are shown in Table S4.

**Figure 7:**
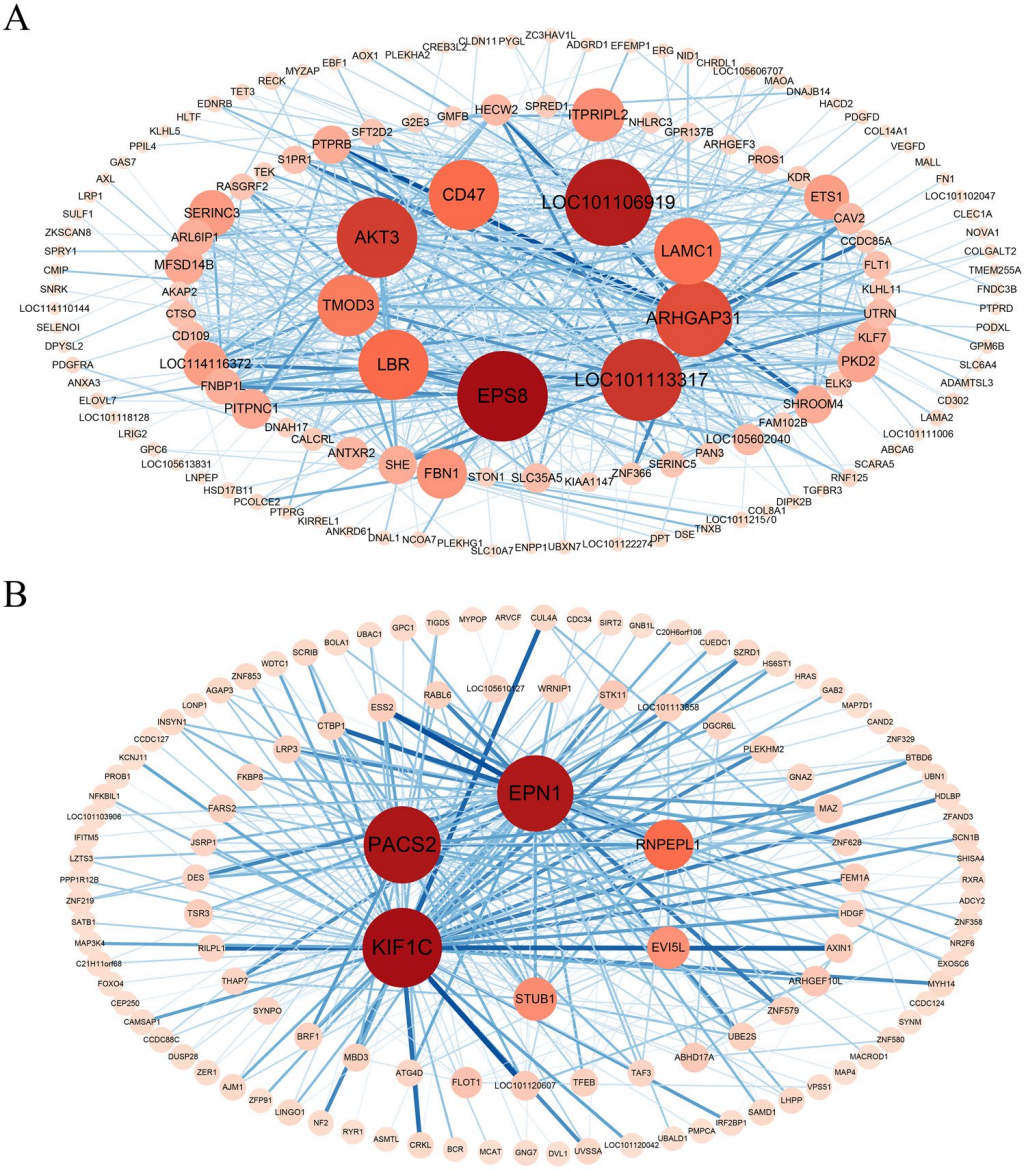
The visualization displays the top 300 links, ranked by correlation coefficients, among transcripts in the blue and turquoise modules. Nodes represent genetic elements. If the symbols representing genes are not known, the transcript IDs are shown. The dimensions and intensity of the red hue of the nodes correspond to their intramodular connection.

Visualization results of the top 300 weight value genes in the blue module showed that there were multiple hub genes in the module, such as LOC101106919 (CYBRD1), LOC101113317, ARHGAP31, EPS8, AKT3, LBR, LAMC1, TMOD3 and CD47, etc. (Fig. 7A). Multiple hub genes were also present in the turquoise (Fig. 7B) module, with the most prominent 3 being EPN1, PACS2 and KIF1C followed by RNPEPL1, EVI5L and STUB1. Hub genes were also found in other modules, such as C12H1orf115 with the highest node values in the brown (Fig. S6) module; FSTL1, PTGFRN, COL5A1 and CTSK in the red (Fig. S6) module; LOC114116925, LOC114118736, LOC114114912 and LOC114118103 in pink module (Figure S6); LOC114116925, LOC114118736 and LOC114114912 in cyan module (Fig. S6); as well as FIT1, LOC114108712, IFIH1, IFI44L and IFIH1 in the black module (Fig. S6), respectively. Details of hub genes are show in Table S4.

## Discussion

### An overview of growth performance, slaughter performance and meat quality of 8-month-old Hu sheep

Animal development is closely related to economic performance. Growth performance information can reveal the production performance of livestock as an important basis for formulating feeding plans in feeding management. Growth performance information not only represents the constitution, carcass structure, growth status, and developmental link of sheep between tissues and organs, but it is also a significant indication for measuring sheep growth and development. Body weight is an essential aspect of sheep production when it comes to growth performance statistics. It is easier to quantify and less error-prone than other growth performance statistics. Weight management considerations are crucial for enhancing productivity and efficiency. According to the findings of this study, the live weight of Hu sheep may represent their carcass structure and growth status to varied degrees, which is an important indication for measuring sheep growth and development.

In this study, there was a substantial association between the growth and slaughterer performance of Hu sheep, both of which were positively connected. This conclusion parallels that of other studies^18–20^. In addition to body length and body height, chest circumference, chest width, chest depth and tube circumference were significantly correlated with live weight and carcass weight. According to several studies, managers should focus on breast and body length data during the production process to estimate sheep growth, which can result in greater economic advantages^18^. This study shows that gender has a greater impact on the live weight and carcass weight of Hu sheep, which is in line with objective laws and consistent with earlier research^18,21,22^. Gender has substantial influence on all growth performance of Hu sheep except chest width.

Many factors influence the meat quality of domestic animals, including species, feeding management methods, food ratio, age, and so on^23–25^. At the same time, there are also large differences among different tissue parts of an individual^26,27^. The pH of sheep meat is a crucial indication of their freshness, which will initially decline and then progressively increase over time after slaughter^28^. At the same time, the stress reaction will result in aberrant pH levels^29^. This study discovered that the backfat thickness, fat content, collagen content, and protein content of ewes were higher than those of rams, albeit this difference was not statistically significant. Research done by Panea Begoña et al.^30^ shows that the moisture, collagen, fat, saturated fatty acids and cholesterol of ewe meat are higher than those of rams. Therefore, ewe meat is more attractive to consumers. The results of this study are similar to those of Panea Begoña et al.^30^.

### Key modules and hub genes associated with the growth and development of 8-month-old Hu sheep

WGCNA is a system biology approach for describing patterns of gene associations among different samples. The correlation among genes is analyzed, which is divided into multiple modules according to the expression patterns of genes, and then analyzed in the modules, thus reducing the amount of calculation and improving the accuracy. We used WGCNA to detect gene association patterns and assess potential interactions among expressed genes. 2 modules (blue and turquoise modules) were determined to be highly correlated with growth and slaughter performance.

The GO enrichment analysis of genes in the blue and brown modules was mainly focuses on the G protein-coupled receptor signaling pathway, the positive regulation of endothelial cell proliferation and other items related to cardiovascular circulatory system development. G protein-coupled receptors (GPCRs) are the largest and most diverse membrane receptors of eukaryotes, regulating a variety of normal biological processes, and playing a role in the pathophysiology of many diseases, which can recognize a variety of ligands and stimuli things^31^. Angiogenesis, the sprouting and branching of new blood vessels from existing vessels, provides circulatory support for tissue development and ischemic tissues, which is a critical process of embryonic development, tissue morphogenesis, pregnancy, wound healing, and tumor development^32^.

A KEGG analysis found that the genes in the blue module were significantly enriched in the PI3K-Akt signaling pathway, the ECM-receptor interaction, focal adhesion and the Rap1 signaling pathway. Protein kinase B (PKB, or Akt) plays a role in cell metabolism, growth, proliferation, and survival^33^. Genes in the brown module are only enriched to the MAPK signaling pathway. The MAPK/ERK pathway (also known as the Ras-Raf-MEK-ERK pathway) is a chain of proteins in cell that communicates a signal from a receptor on its surface to the DNA in its nucleus, which regulates processes such as cell proliferation, cell differentiation, and cell deaths of eukaryotes from yeast to humans^34–36^.

Genes in the turquoise module mostly concentrated on Go items related to energy metabolism and small-molecule metabolism strips. Pathways including dilated cardiomyopathy, hypertrophic cardiomyopathy, carbon metabolism, and the oxytocin signaling pathway were substantially more prevalent in genes in the turquoise module.

Genes in the red module exhibited a significant enrichment in GO terms associated with several aspects of development, such as skeletal system development, cartilage development, and extracellular matrix organization. KEGG enrichment analysis examines the pathways related to protein digestion and absorption as well as ECM-receptor interaction. The following hub genes were identified: FSTL1, PTGFRN, COL5A1, and CTSK.

Genes inside the black module have a higher probability of participating in biological responses to stimuli and immune defenses against viruses. The identified hub genes are FIT1, LOC114108712, IFIH1, IFI44L, and IFIH1.

### Supplementary Information

Supplementary Figure S1.

Supplementary Figure S2.

Supplementary Figure S3.

Supplementary Figure S4.

Supplementary Figure S5.

Supplementary Figure S6.

Supplementary Table S1.

Supplementary Table S2.

Supplementary Table S3.

Supplementary Table S4.

## Data Availability

The sequence data has been deposited into Sequence Read Archive Database of National Center for Biotechnology Information (NCBI) with the accession number of SRP428602.
