# Supplementary material for: Weighted gene co-expression network analysis reveals genes related to growth performance in Hu sheep
Source: Sci Rep. 2024 Jun 6;14:13043. doi: 10.1038/s41598-024-63850-x (PMC11156982; doi:10.1038/s41598-024-63850-x)
Supplement: Supplementary file 1 — Supplementary Figure S1. [file 41598_2024_63850_MOESM1_ESM.docx]

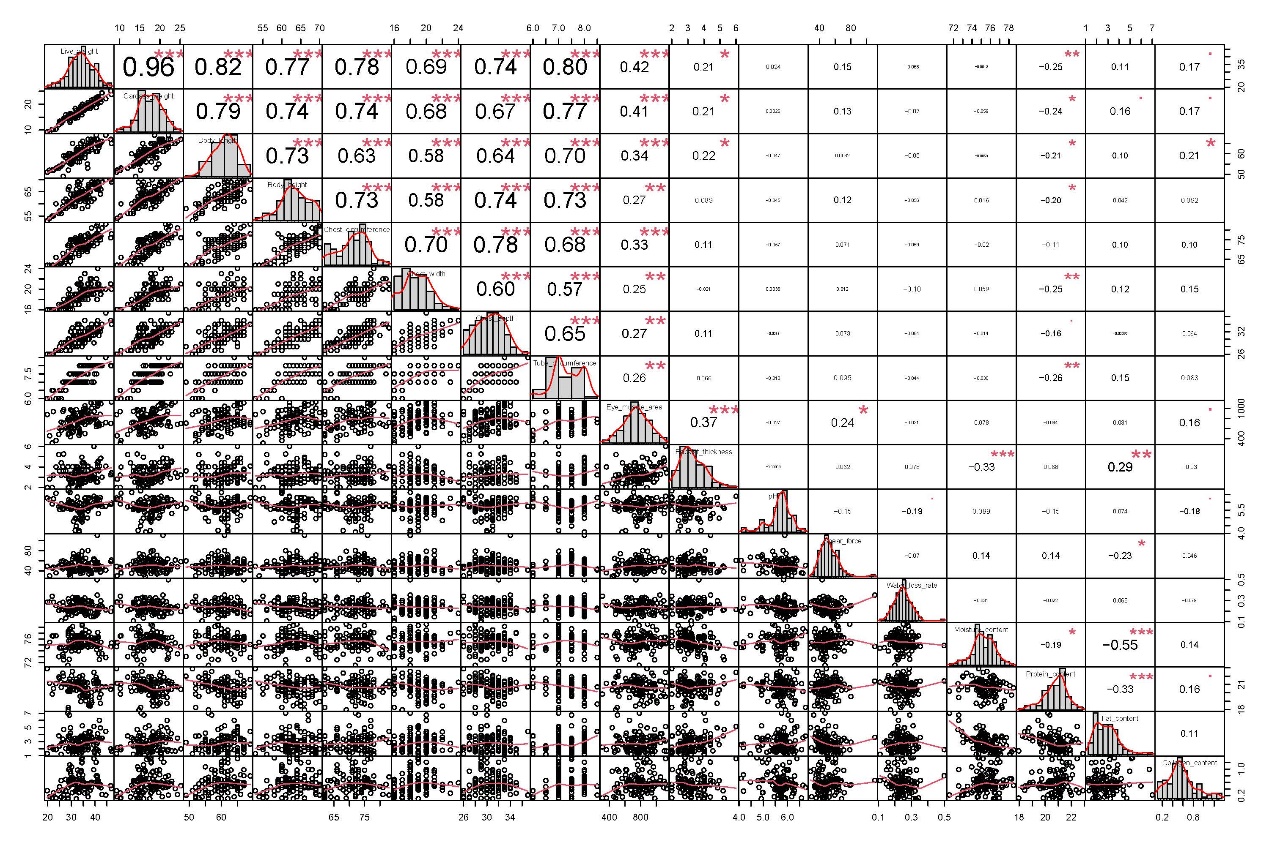


**Figure. S1.** Correlation analysis among on the growth performance & slaughter performance and meat quality performance. The scatter plots are shown in the bottom left corner, the correlation-significance plots among features are displayed in the upper right corner, and the data distribution map is displayed in the center. *, **, and *** represent significance at ***P*** < 0.05, 0.01, and 0.001, respectively.
