# Supplementary material for: Weighted gene co-expression network analysis reveals genes related to growth performance in Hu sheep
Source: Sci Rep. 2024 Jun 6;14:13043. doi: 10.1038/s41598-024-63850-x (PMC11156982; doi:10.1038/s41598-024-63850-x)
Supplement: Supplementary file 2 — Supplementary Figure S2. [file 41598_2024_63850_MOESM2_ESM.docx]

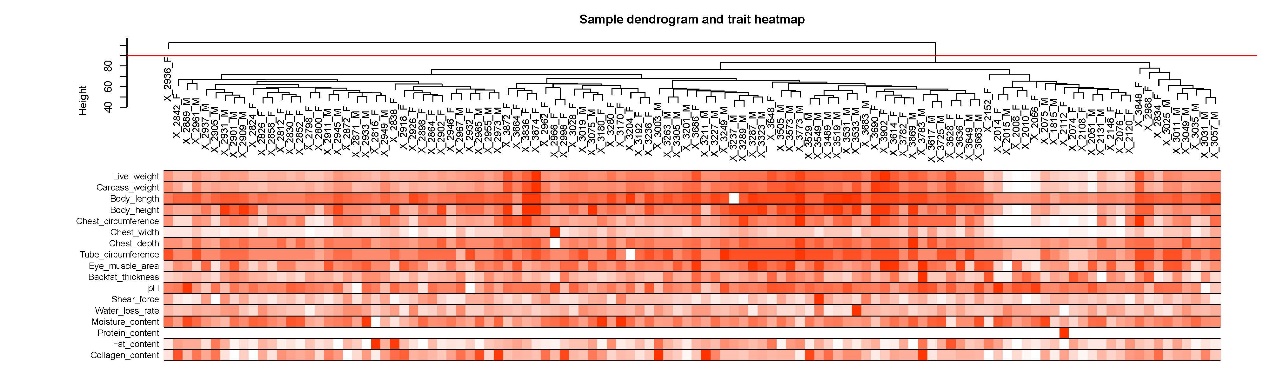


**Figure. S2** Sample clustering to detect outliers. Cluster all samples and show that there is an outlier.
