# Supplementary material for: Weighted gene co-expression network analysis reveals genes related to growth performance in Hu sheep
Source: Sci Rep. 2024 Jun 6;14:13043. doi: 10.1038/s41598-024-63850-x (PMC11156982; doi:10.1038/s41598-024-63850-x)
Supplement: Supplementary file 3 — Supplementary Figure S3. [file 41598_2024_63850_MOESM3_ESM.docx]

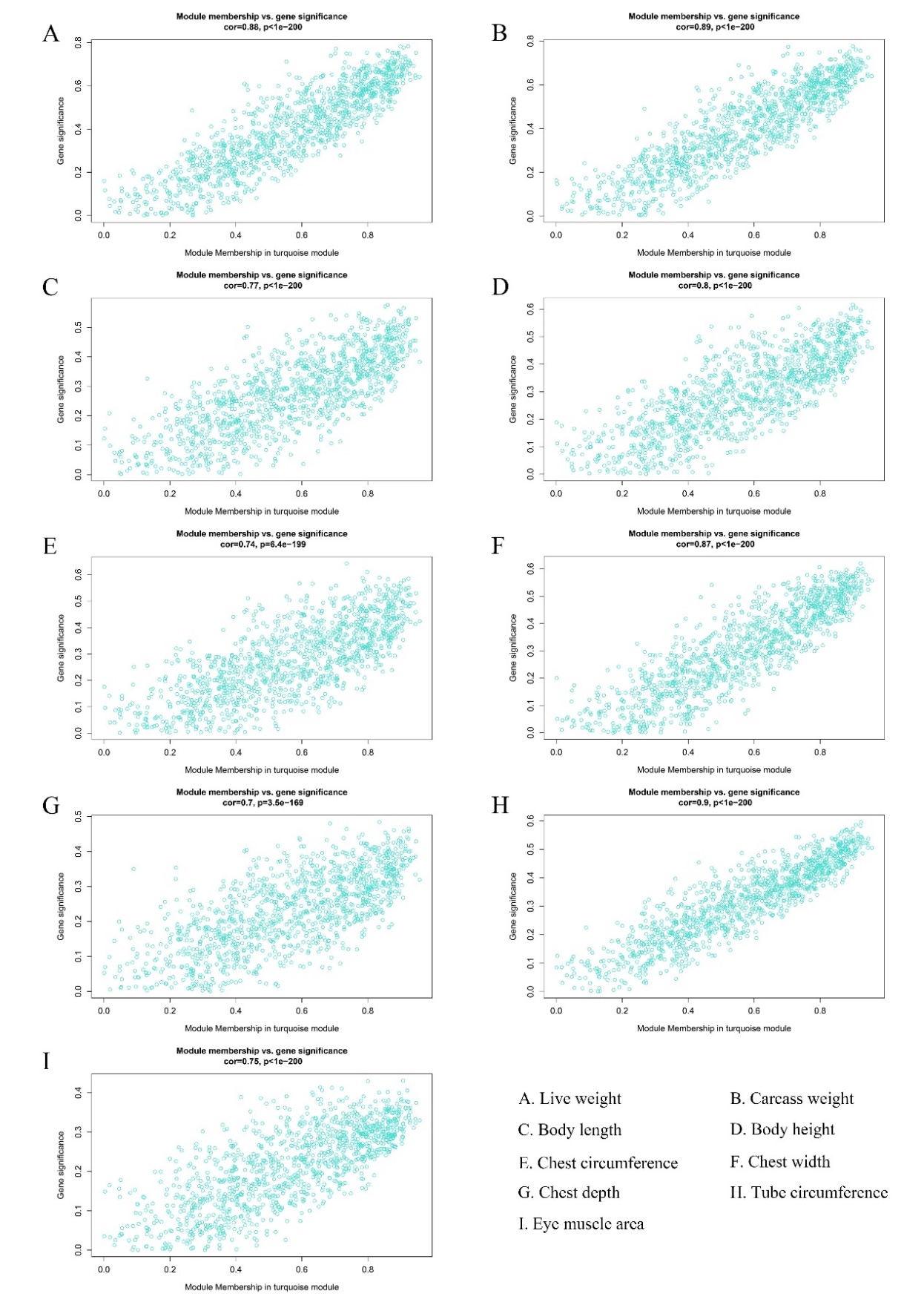


**Figure. S3.** A scatterplot of gene significance (GS) for slaughter performance *vs.* module membership (MM) in turquoise module. Each point in the figure represents a gene, the abscissa value represents the correlation between the gene and the module, and the ordinate value represents the correlation between the gene and the phenotypic trait.


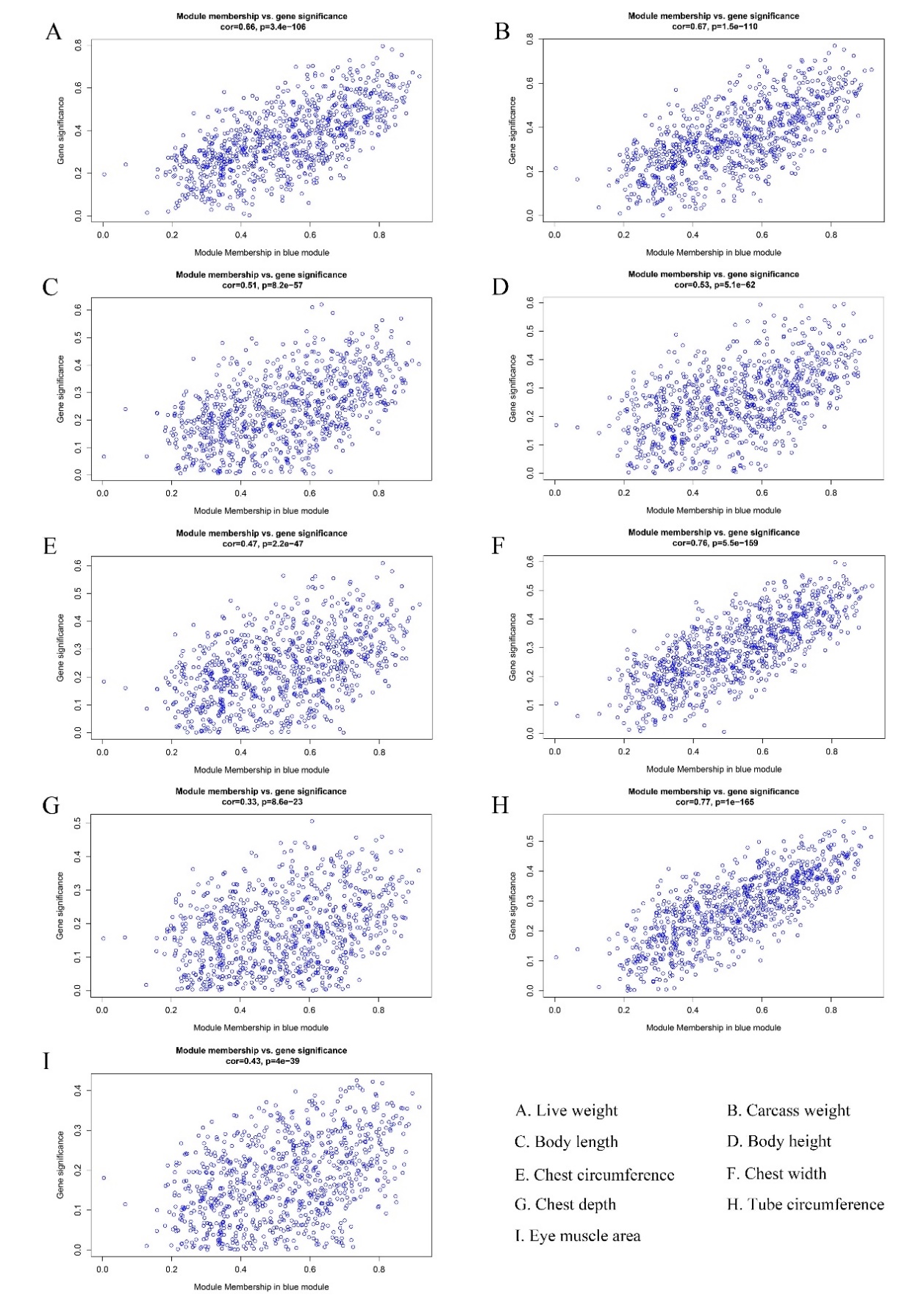


**Figure. S3.** A scatterplot of gene significance (GS) for slaughter performance *vs.* module membership (MM) in blue module. Each point in the figure represents a gene, the abscissa value represents the correlation between the gene and the module, and the ordinate value represents the correlation between the gene and the phenotypic trait.


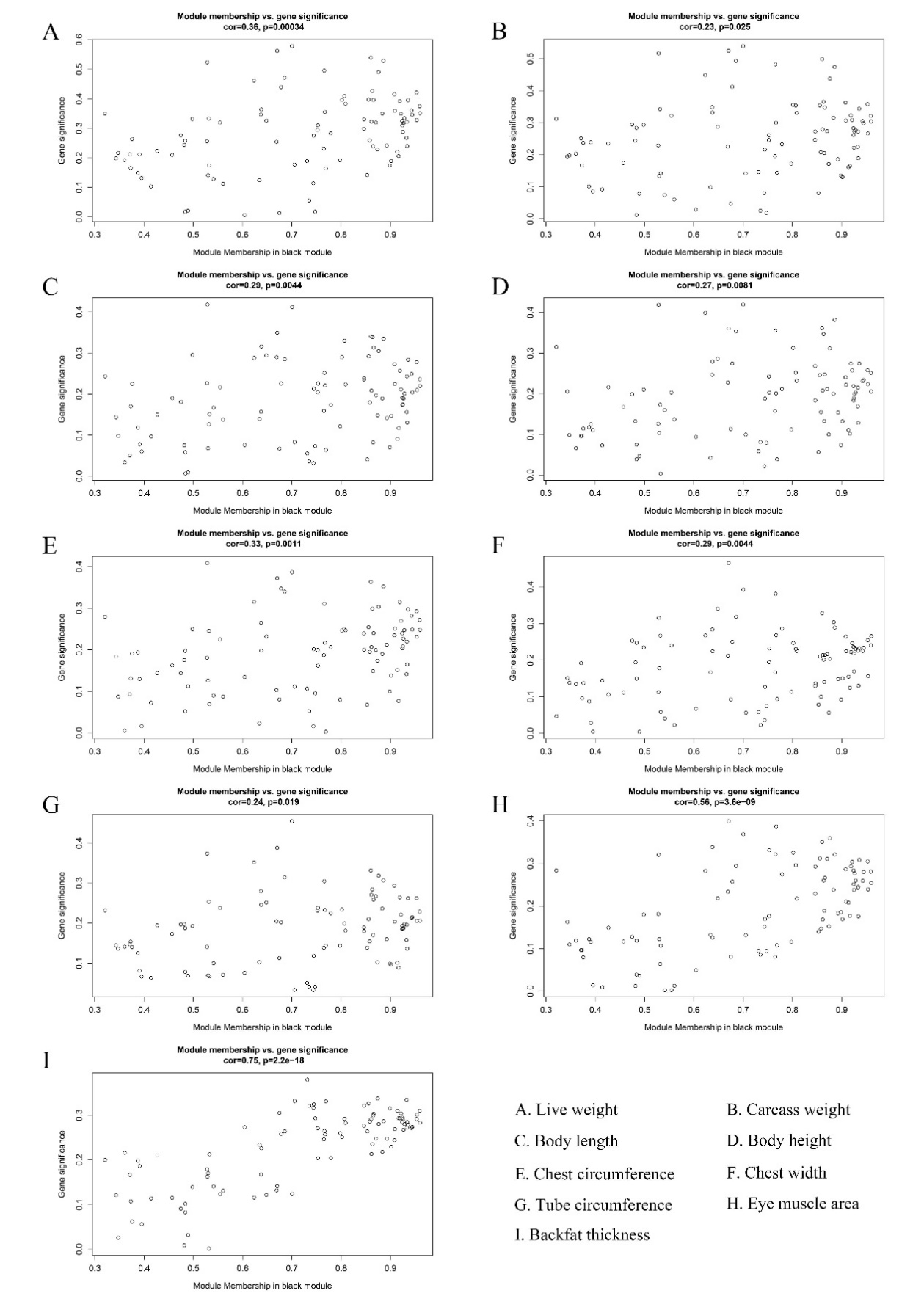


**Figure. S3.** A scatterplot of gene significance (GS) for slaughter performance *vs.* module membership (MM) in black module. Each point in the figure represents a gene, the abscissa value represents the correlation between the gene and the module, and the ordinate value represents the correlation between the gene and the phenotypic trait.


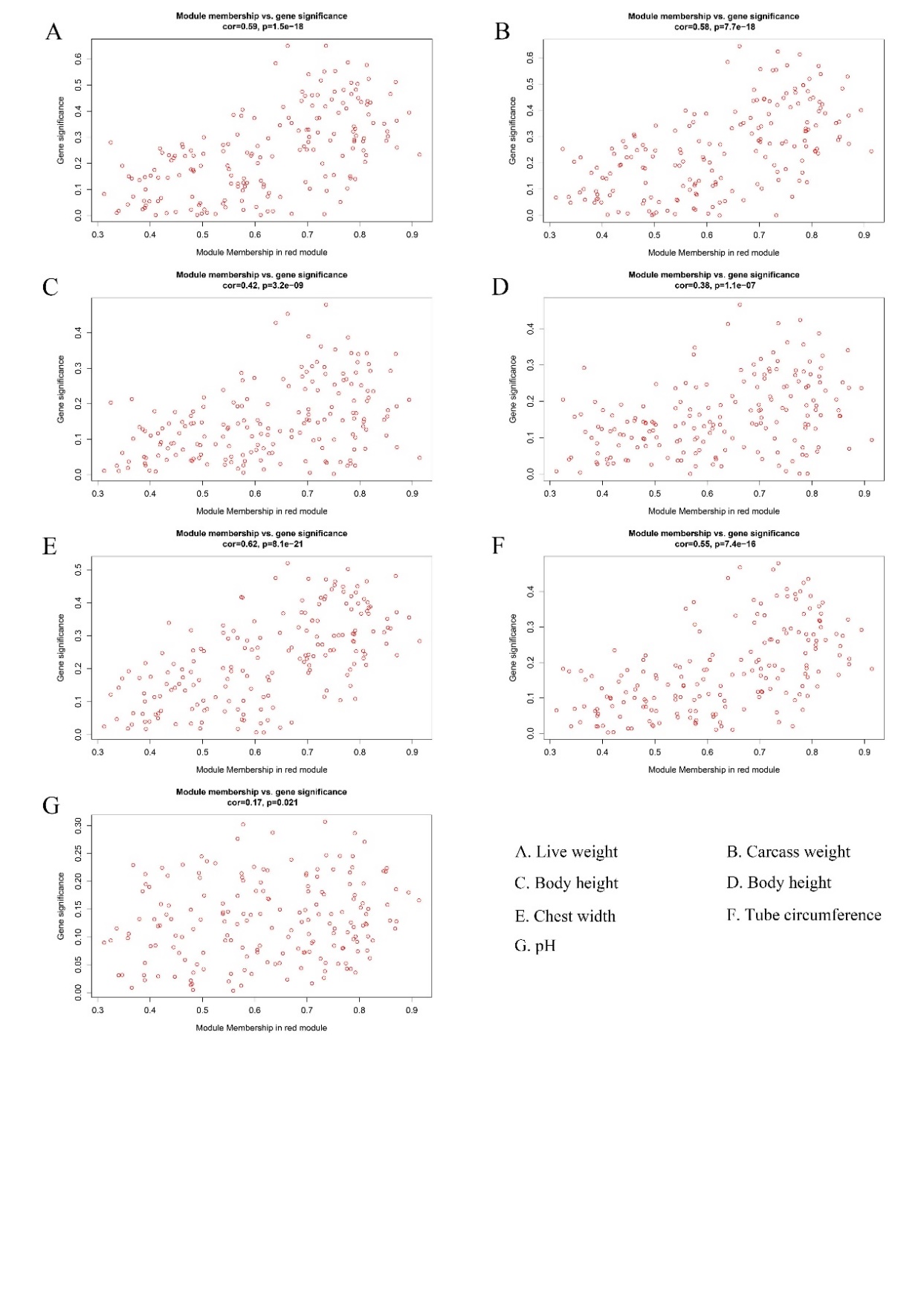


**Figure. S3.** A scatterplot of gene significance (GS) for slaughter performance *vs.* module membership (MM) in red module. Each point in the figure represents a gene, the abscissa value represents the correlation between the gene and the module, and the ordinate value represents the correlation between the gene and the phenotypic trait.


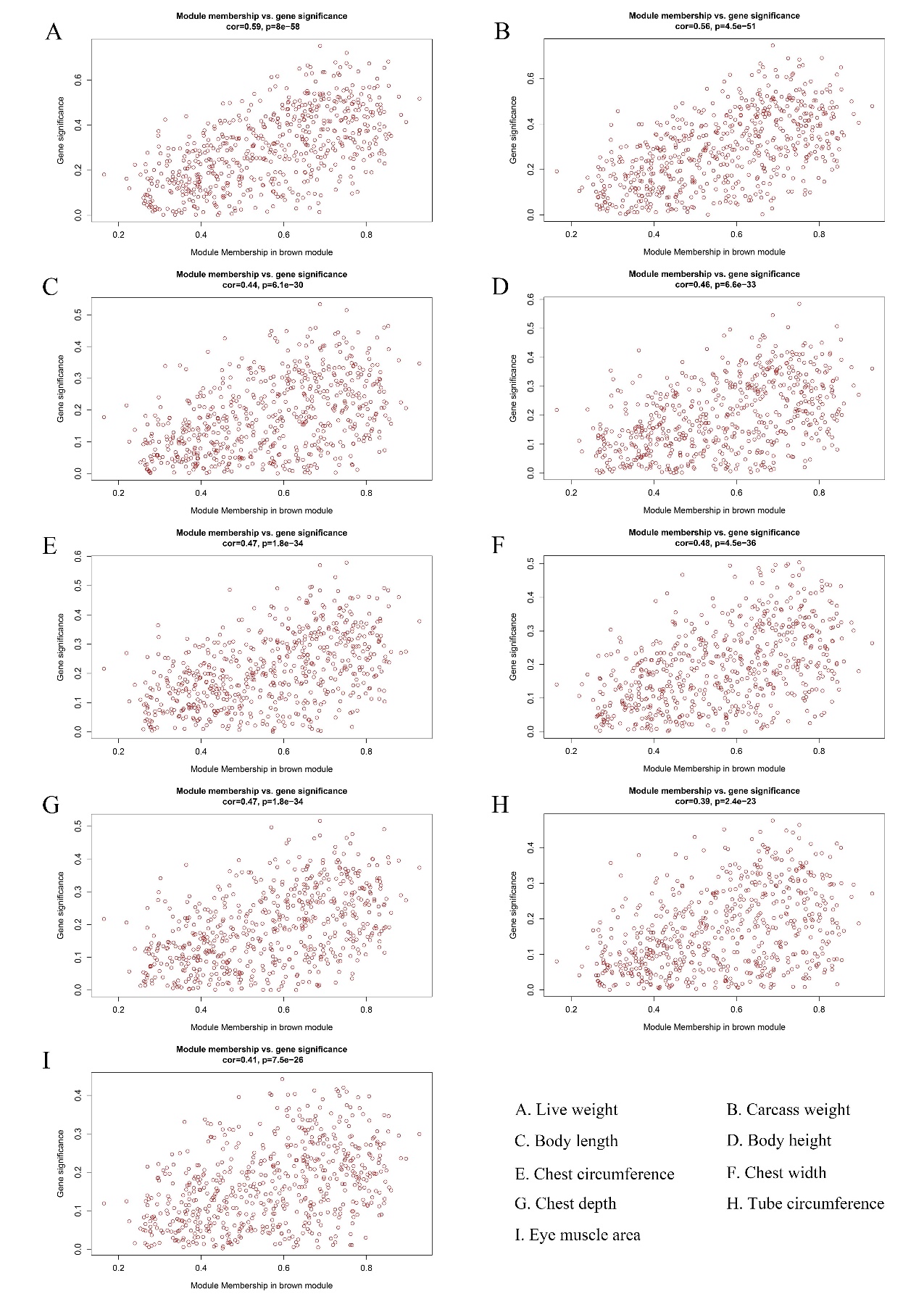


**Figure. S3.** A scatterplot of gene significance (GS) for slaughter performance *vs.* module membership (MM) in brown module. Each point in the figure represents a gene, the abscissa value represents the correlation between the gene and the module, and the ordinate value represents the correlation between the gene and the phenotypic trait.


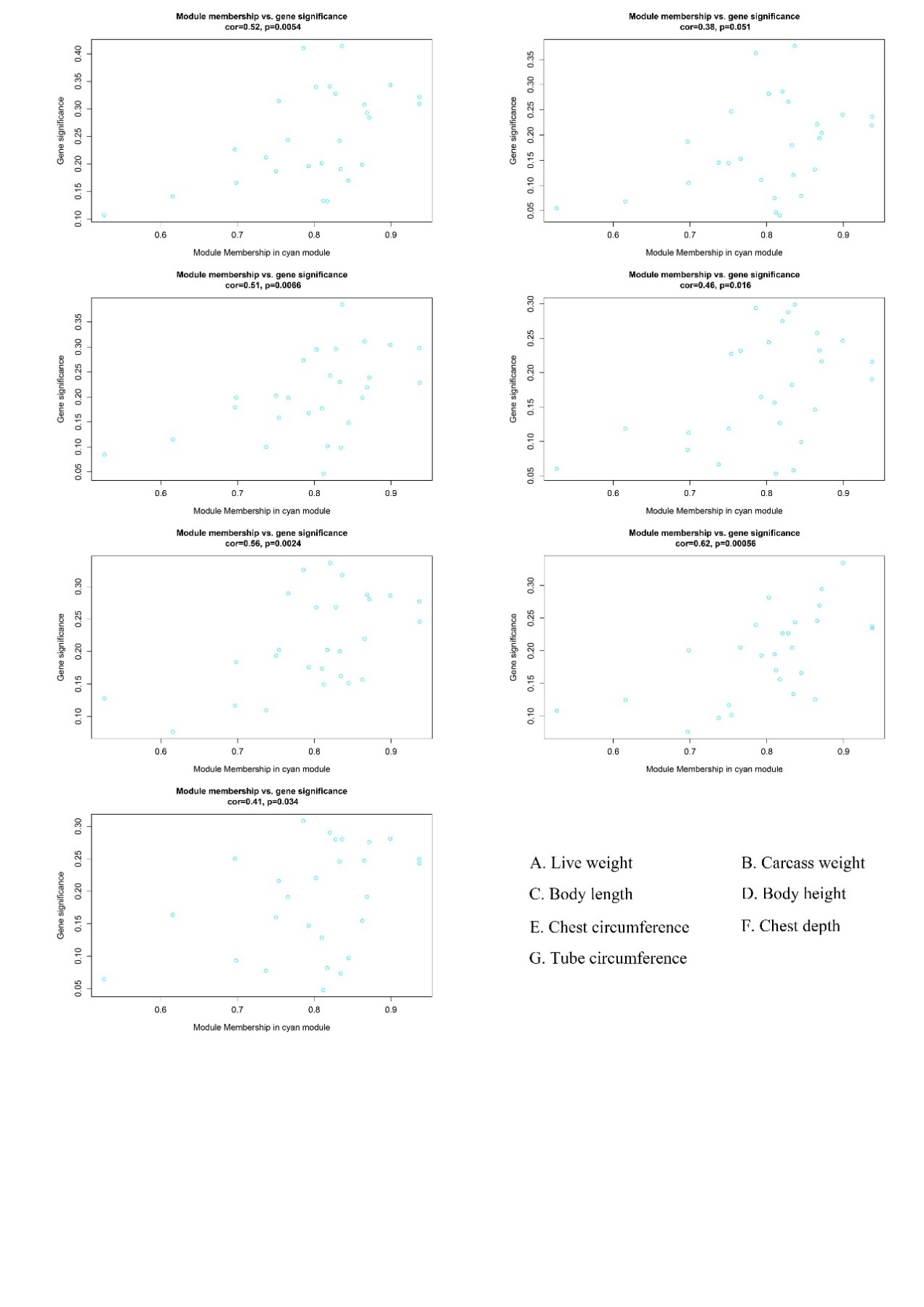


**Figure. S3.** A scatterplot of gene significance (GS) for slaughter performance *vs.* module membership (MM) in cyan module. Each point in the figure represents a gene, the abscissa value represents the correlation between the gene and the module, and the ordinate value represents the correlation between the gene and the phenotypic trait.


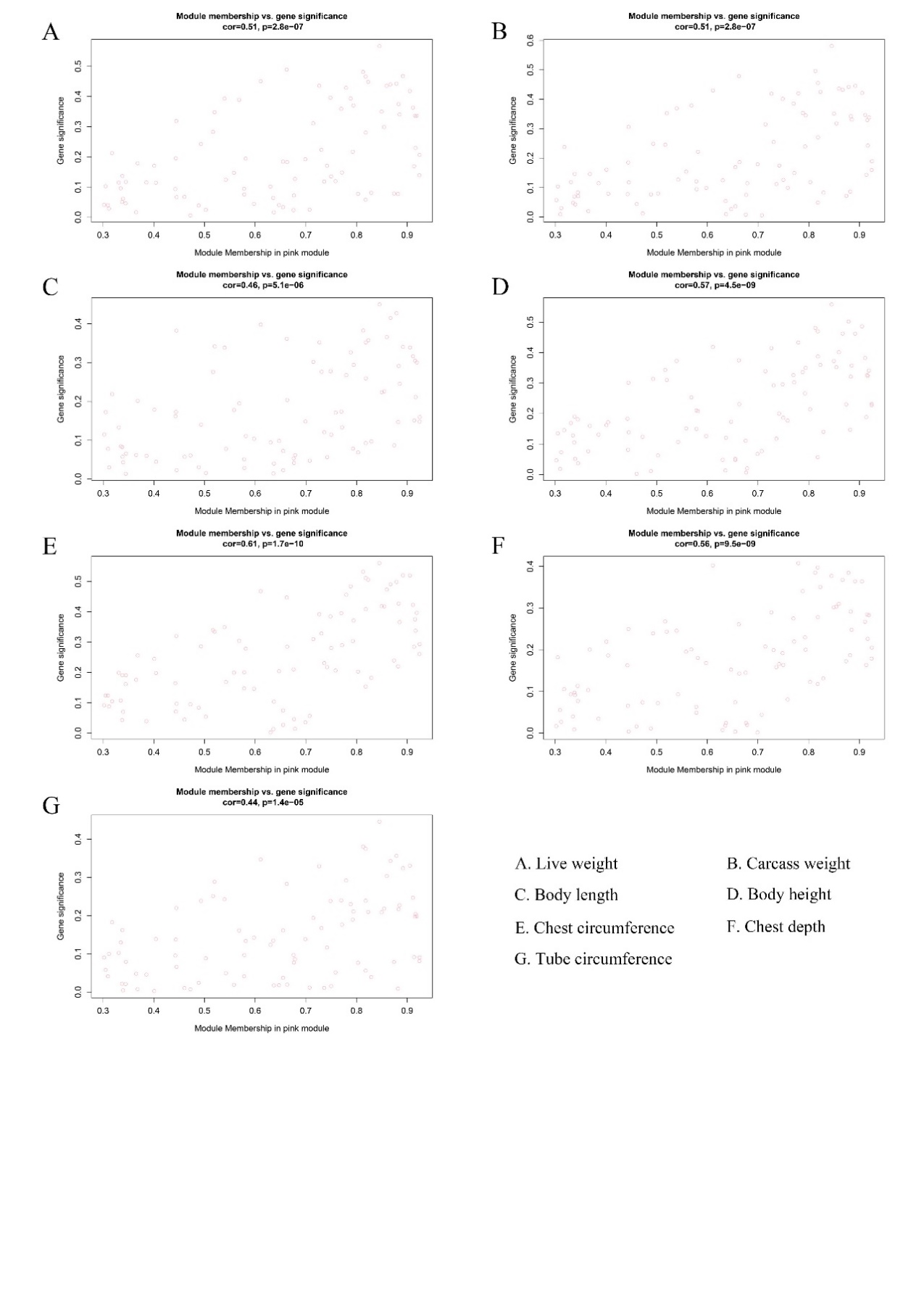


**Figure. S3.** A scatterplot of gene significance (GS) for slaughter performance *vs.* module membership (MM) in pink module. Each point in the figure represents a gene, the abscissa value represents the correlation between the gene and the module, and the ordinate value represents the correlation between the gene and the phenotypic trait.
