# Supplementary material for: Weighted gene co-expression network analysis reveals genes related to growth performance in Hu sheep
Source: Sci Rep. 2024 Jun 6;14:13043. doi: 10.1038/s41598-024-63850-x (PMC11156982; doi:10.1038/s41598-024-63850-x)
Supplement: Supplementary file 4 — Supplementary Figure S4. [file 41598_2024_63850_MOESM4_ESM.docx]

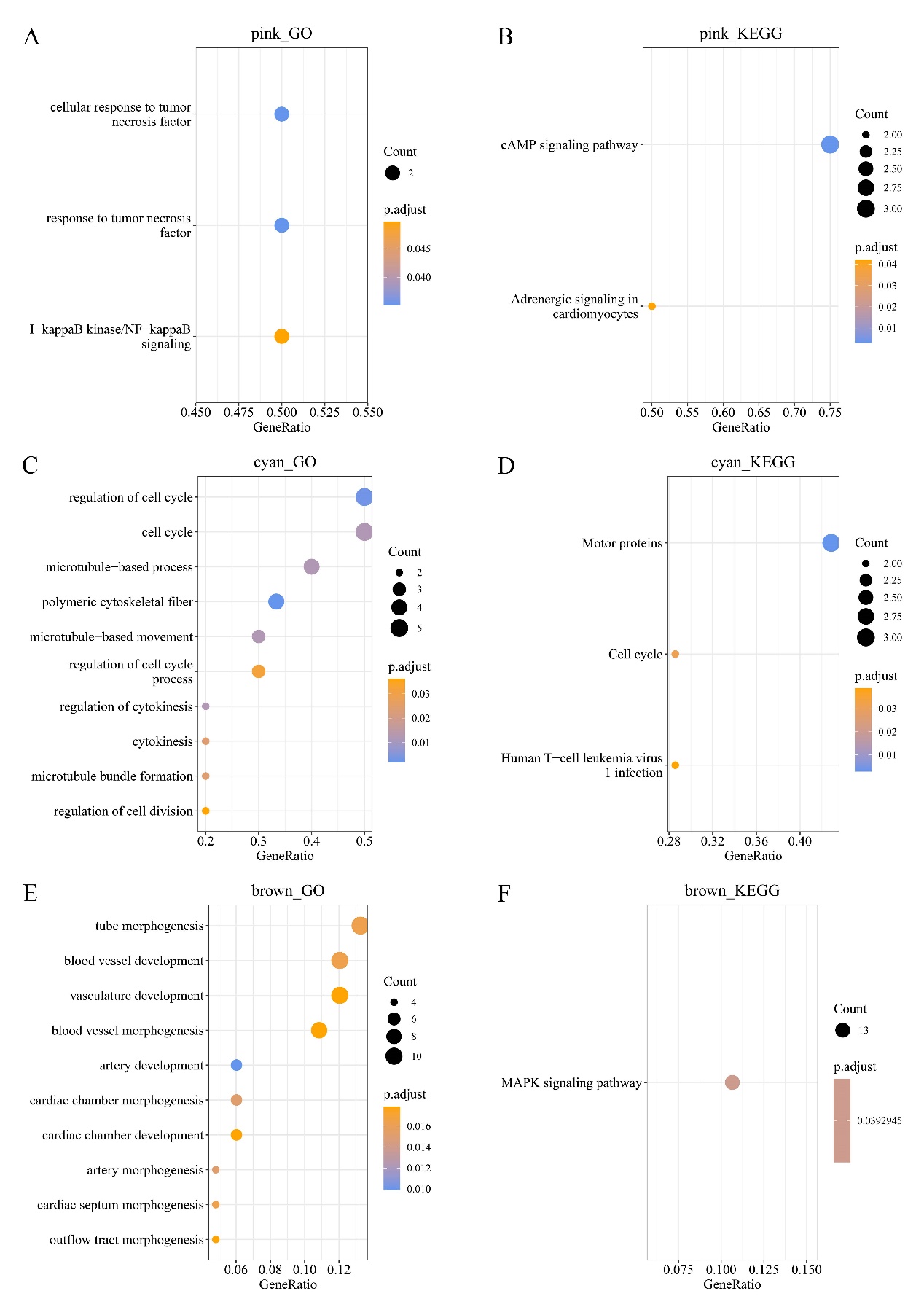

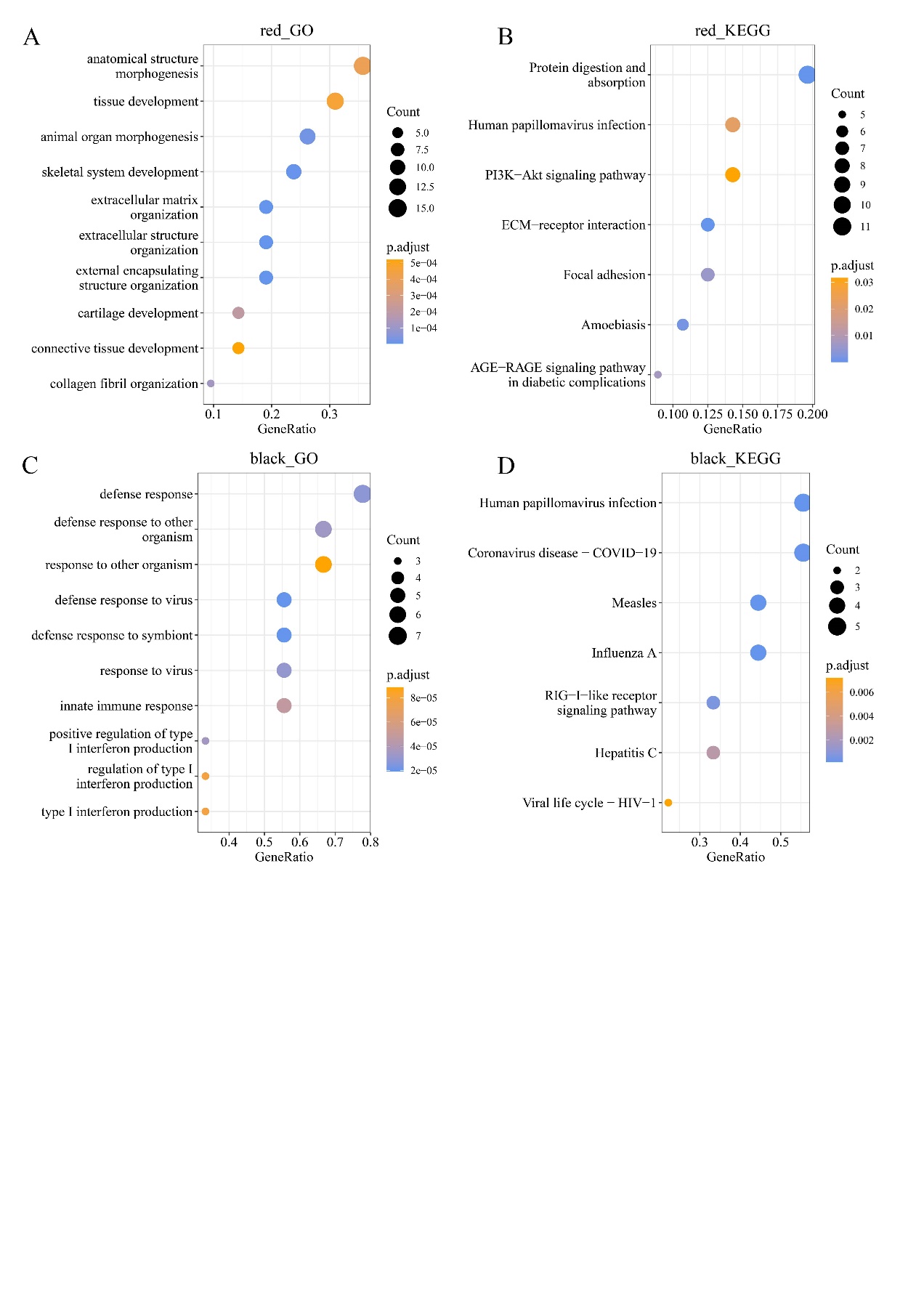


**Figure. S4.** Functional annotation analysis of genes within the modules of pink, cyan, brown, red and black.
