# Supplementary figures and images for: Weighted gene co-expression network analysis reveals genes related to growth performance in Hu sheep
Source: Sci Rep. 2024 Jun 6;14:13043. doi: 10.1038/s41598-024-63850-x (PMC11156982; doi:10.1038/s41598-024-63850-x)

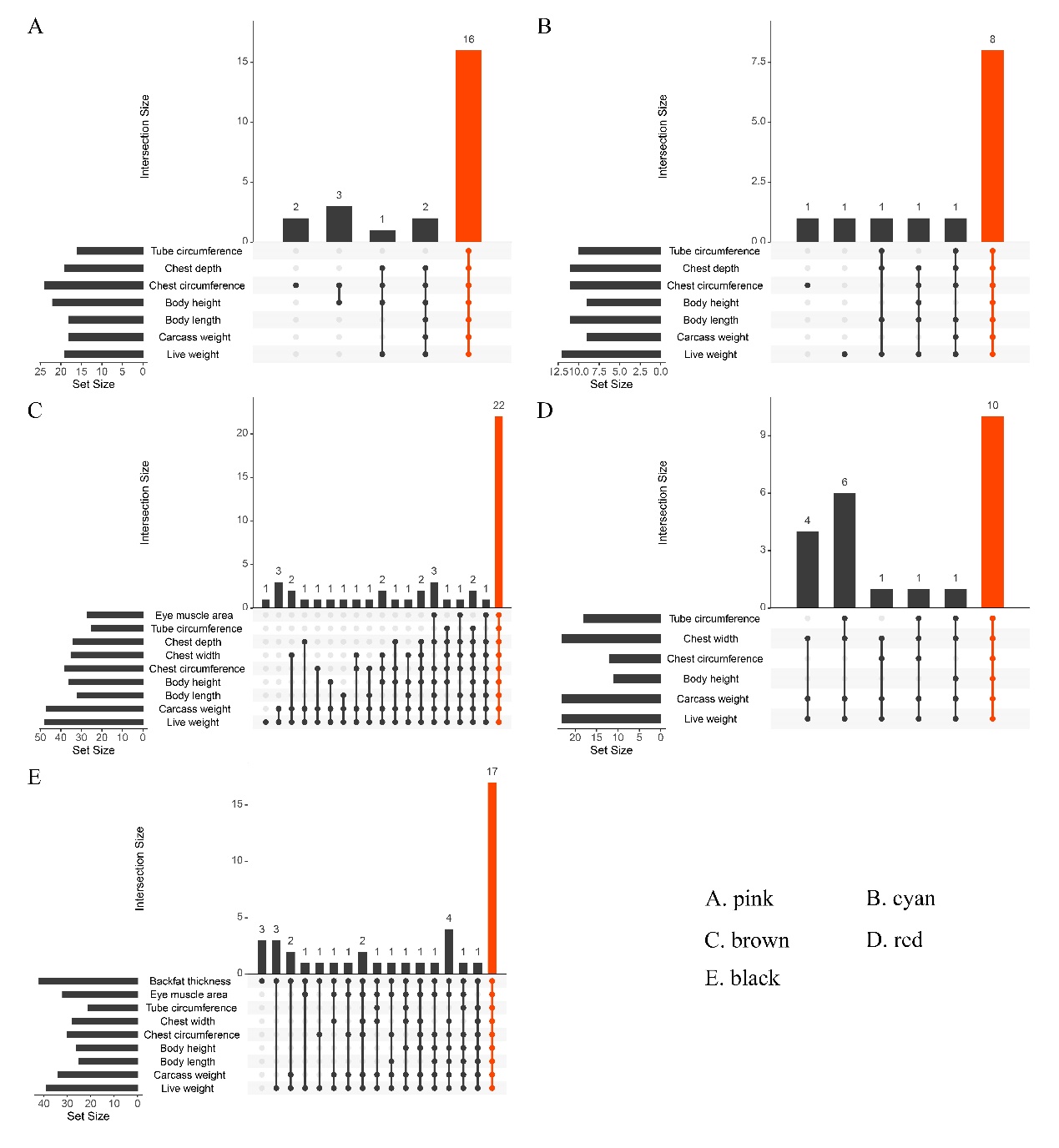


**Figure. S5.** Overlapped genes of slaughter performance in the pink, cyan, brown, red, black modules.

Supplement: Supplementary file 5 — Supplementary Figure S5. [file 41598_2024_63850_MOESM5_ESM.docx]
