# Supplementary material for: Weighted gene co-expression network analysis reveals genes related to growth performance in Hu sheep
Source: Sci Rep. 2024 Jun 6;14:13043. doi: 10.1038/s41598-024-63850-x (PMC11156982; doi:10.1038/s41598-024-63850-x)
Supplement: Supplementary file 6 — Supplementary Figure S6. [file 41598_2024_63850_MOESM6_ESM.docx]

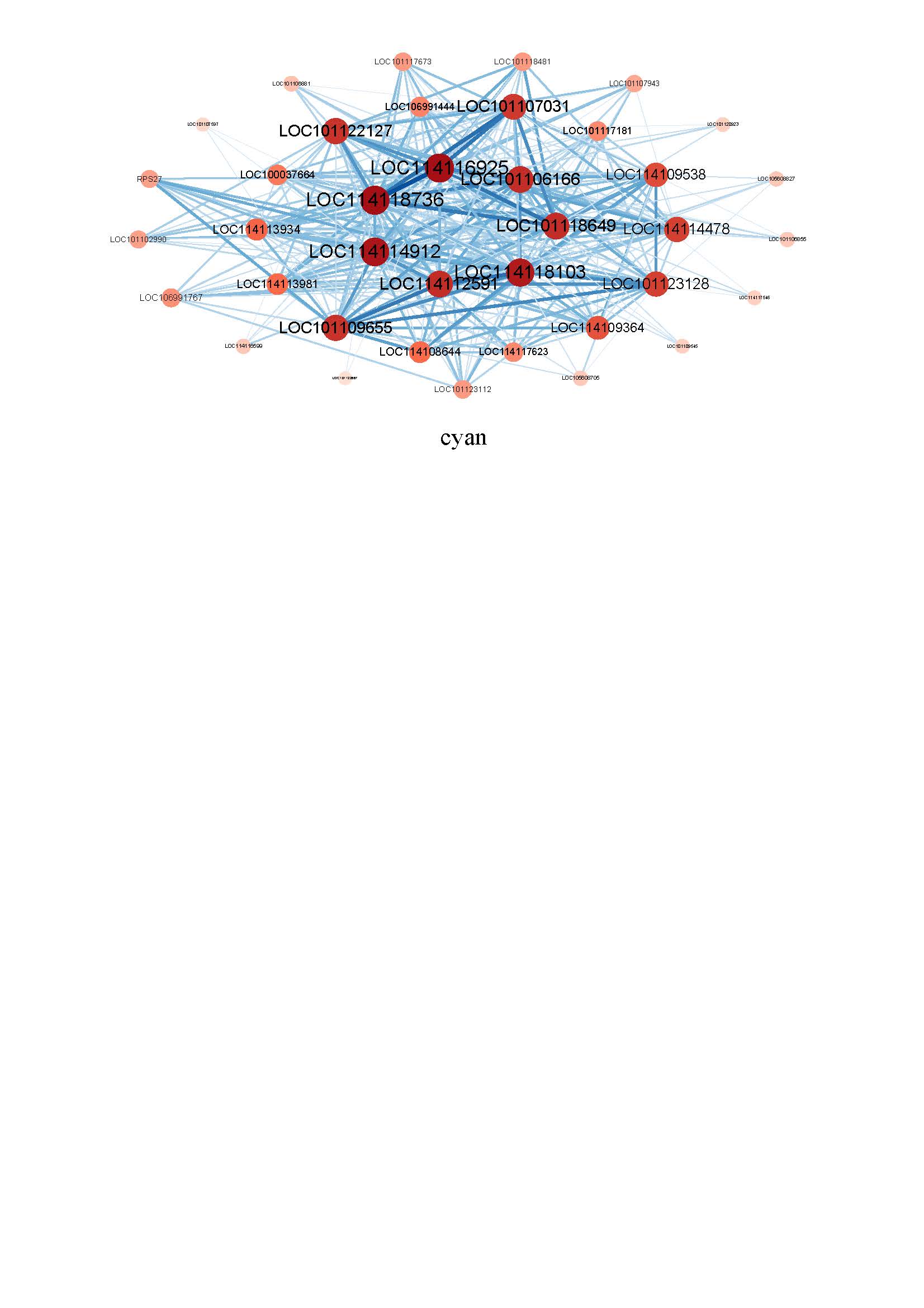

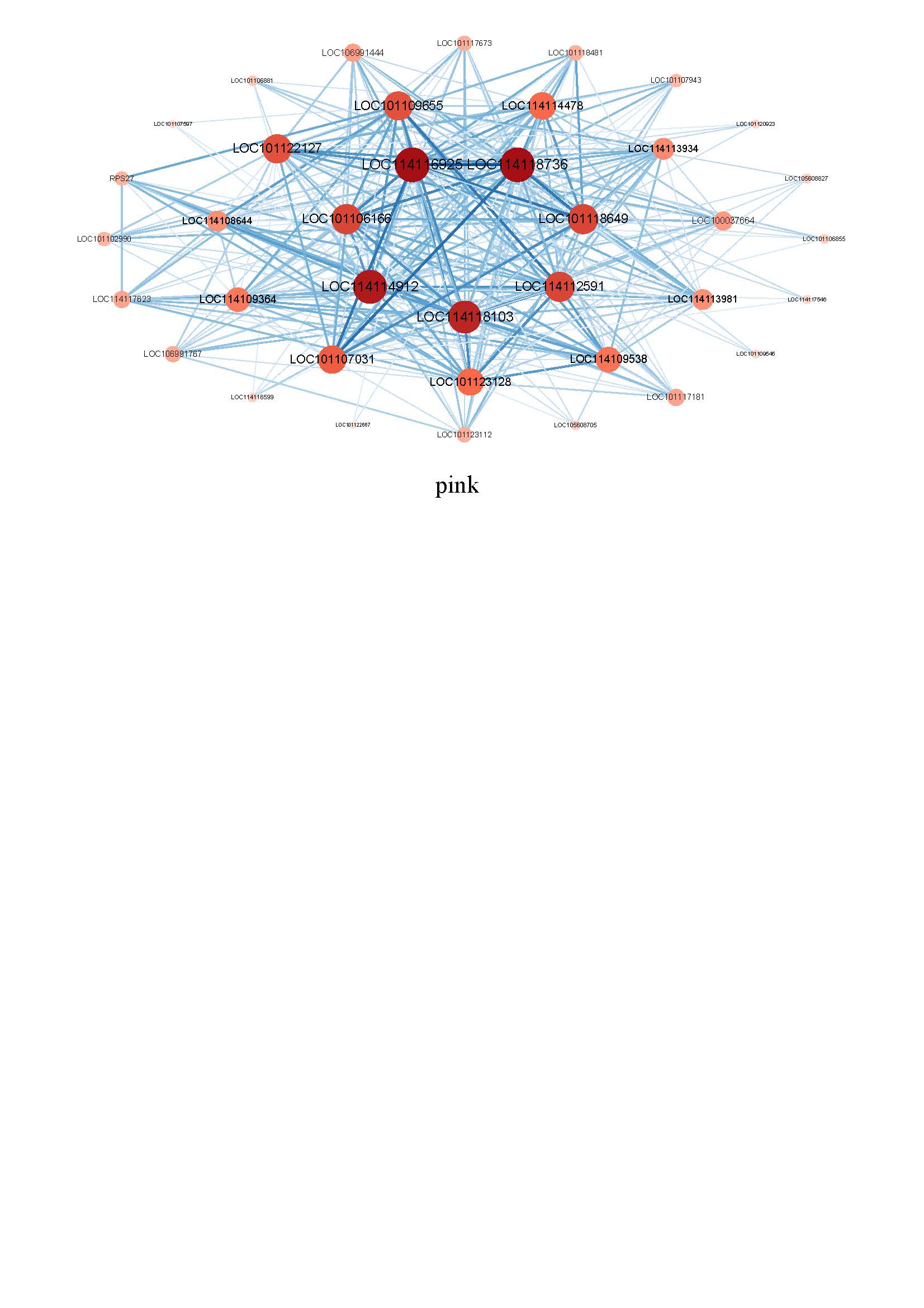

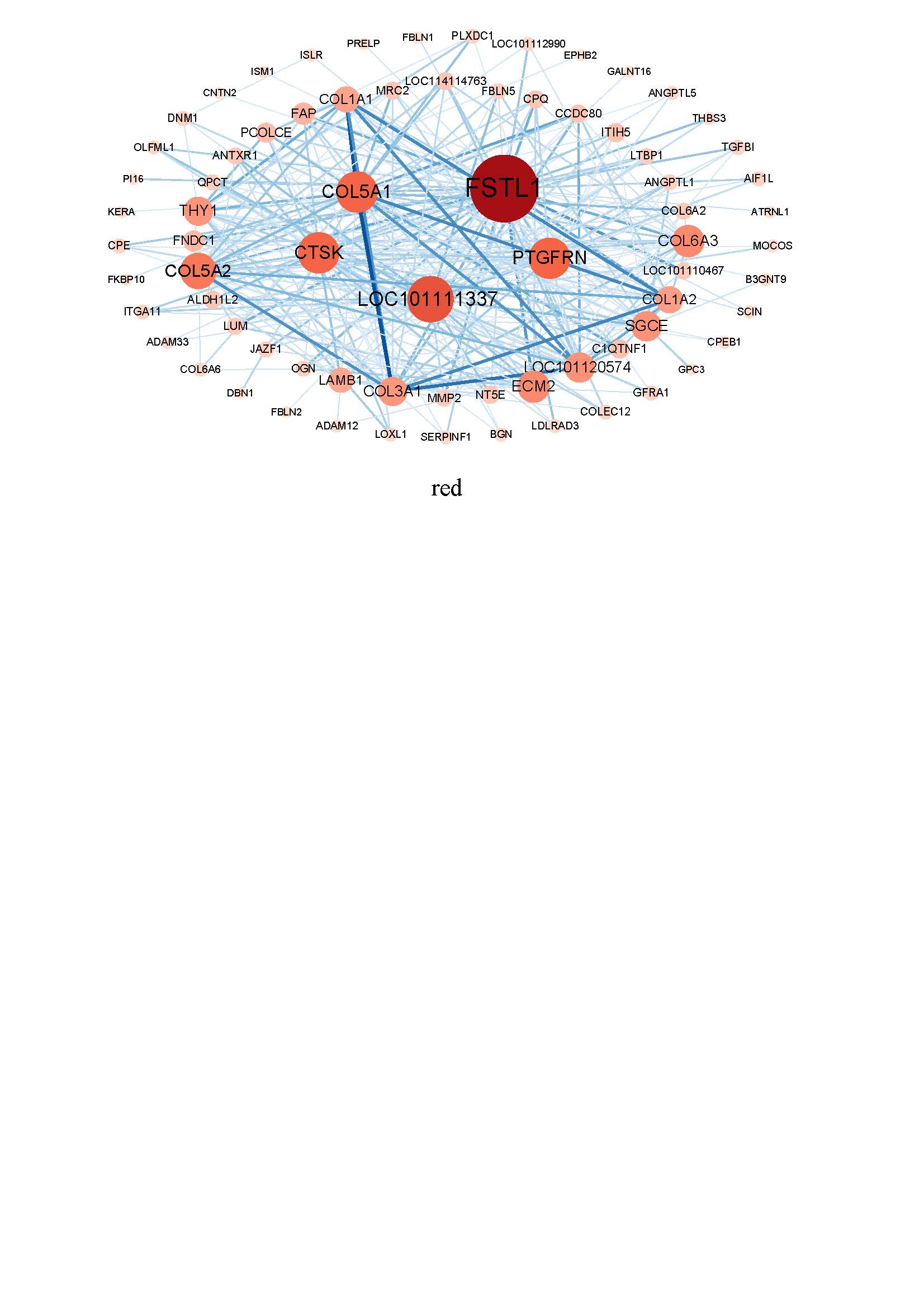

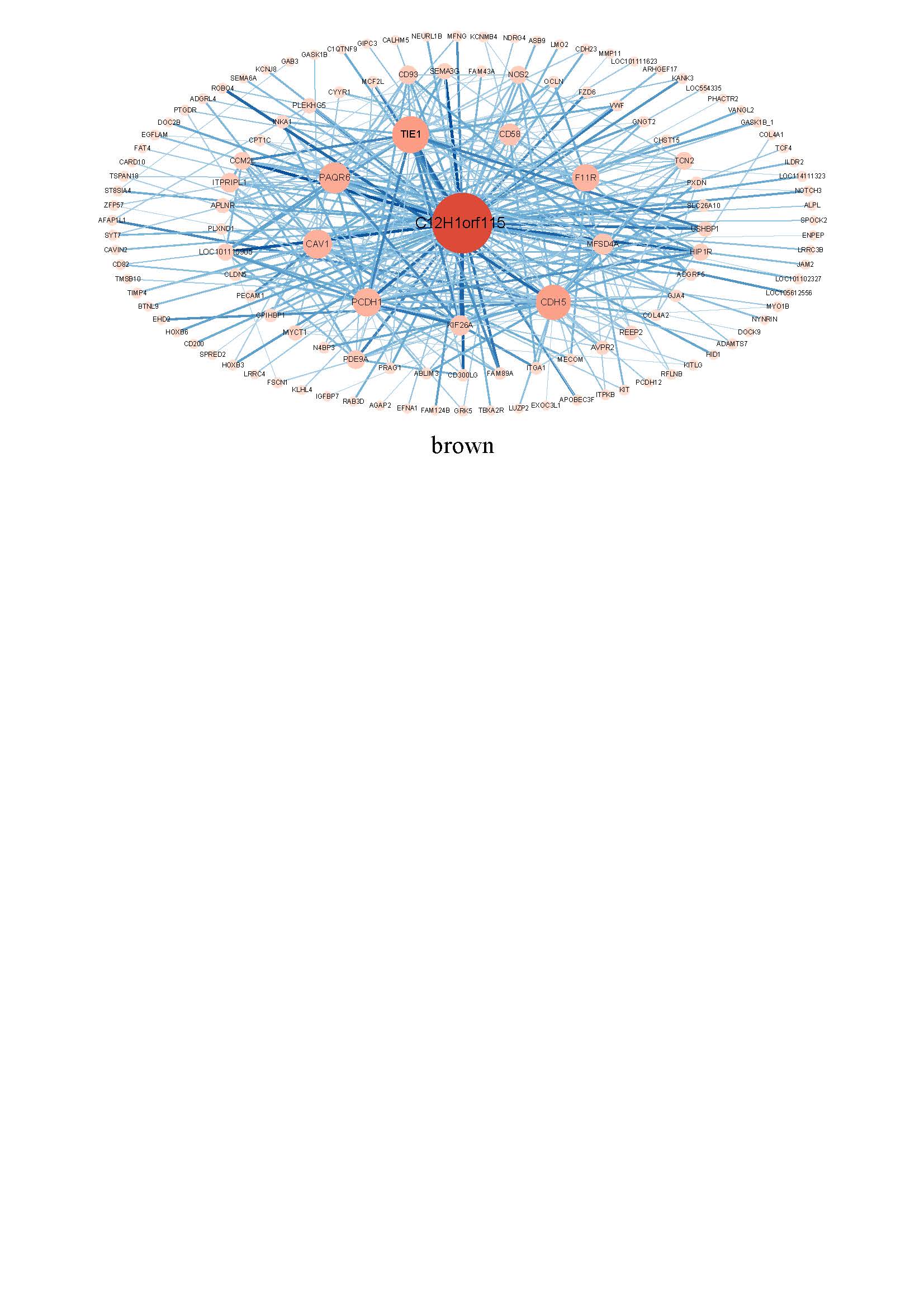

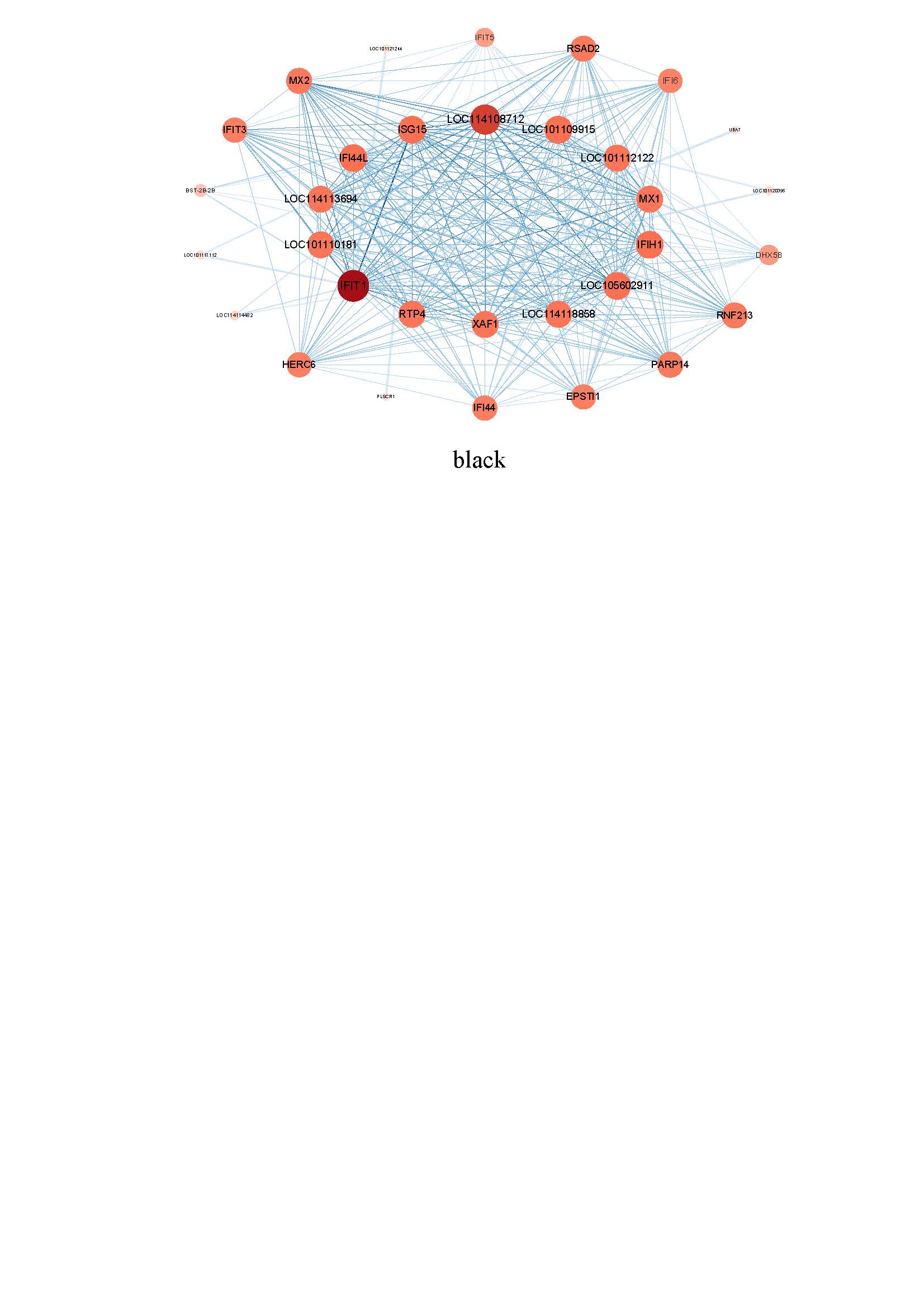


**Figure. S6.** The visualization of brown, red, pink, cyan, and black modules show the top 300 transcript correlation coefficient-sorted links.
